# Endometrial Cancer Is Associated with Altered Metabolism and Composition of Fatty Acids

**DOI:** 10.3390/ijms26073322

**Published:** 2025-04-02

**Authors:** Yelyzaveta Razghonova, Adriana Mika, Monika Czapiewska, Agata Stanczak, Paulina Zygowska, Dariusz Grzegorz Wydra, Tomasz Sledzinski, Anna Abacjew-Chmylko

**Affiliations:** 1 Department of Pharmaceutical Biochemistry, Faculty of Pharmacy, Medical University of Gdansk, 80-211 Gdansk, Poland; yelyzaveta.razghonova@gumed.edu.pl (Y.R.); monika.czapiewska99@gumed.edu.pl (M.C.); tomasz.sledzinski@gumed.edu.pl (T.S.); 2 Department of Environmental Analytics, Faculty of Chemistry, University of Gdansk, 80-308 Gdansk, Poland; 3 Department of Obstetrics and Gynecology, Gynecological Oncology and Endocrinological Gynecology, University Clinical Center, 80-952 Gdansk, Poland; astanczak@uck.gda.pl (A.S.); pzygowska@uck.gda.pl (P.Z.); dwydra@icloud.com (D.G.W.); 4 Department of Obstetrics and Gynaecology and Gynecological Oncology and Endocrinological Gynecology, Medical University of Gdansk, 80-952 Gdansk, Poland

**Keywords:** fatty acid transport, endometrial cancer, de novo lipogenesis, metabolic reprogramming, stage-specific changes

## Abstract

Endometrial cancer (EC) is a complex gynecologic malignancy that requires a deeper understanding of its molecular basis to improve therapeutic strategies. In this study, we investigated the role of fatty acid (FA) reprogramming in the progression of EC. We analyzed FA profiles to identify the stage-specific changes and gene expression profiles of key enzymes involved in FA synthesis, desaturation, elongation, transport, and oxidation at different stages of EC. Our results show that EC tissues have lower levels of saturated FA and branched-chain FA, higher levels of very long-chain FA, n-3 polyunsaturated FA (PUFA), and monounsaturated FA, with the exception of myristoleic acid. The differences in n-6 PUFA were inconsistent. Gene expression analysis revealed the upregulation of key enzymes controlling de novo FA synthesis, including *ACACA*, *FASN*, *SCD1*, and *ELOVL1*. In contrast, the expression of genes related to FA transport in the cell and β-oxidation was downregulated. The expression of some genes related to PUFA metabolism was upregulated, while others were downregulated. These results demonstrate a reprogramming of lipid metabolism in EC tissues and suggest potential targets for novel therapeutic interventions in EC.

## 1. Introduction

Endometrial cancer (EC) is a significant concern in gynecologic oncology, as its incidence has increased significantly, particularly in developed countries. Moreover, the mortality rate associated with this disease has hardly improved, despite advances in diagnostic techniques and therapeutic interventions. The incidence and mortality rate of EC is expected to increase 1.6-fold by 2050 [1]. By 2022, it is expected to be the sixth-most common cancer among women worldwide [2,3]. These worrying statistics highlight the urgent need to tackle the increasing burden of EC. Surgery is the foundation of EC treatment; however, adjuvant therapy (radiotherapy and/or chemotherapy) is indicated in intermediate- and high-risk patients to improve treatment efficacy. Accordingly, a deeper understanding of the molecular basis of EC development is crucial for the progress of personalized medicine in the treatment of this neoplasm.

Several molecular markers are associated with the development of this malignancy, including *TP53* mutations, altered Wnt signaling, disruption of the mTOR pathway, decreased expression of estrogen and progesterone receptors, and abnormal expression of the L1 cell adhesion molecule (L1CAM) [4]. Metabolic reprogramming within EC cells is increasingly recognized as a critical factor in tumorigenesis and disease progression. A notable feature of this reprogramming is an increased uptake of glucose and the production of lactate, which are indicative of increased glycolysis—a common adaptation seen in cancer cells. Furthermore, the dysregulation of amino acid metabolism, particularly involving branched-chain amino acids and glutamine, has been associated with tumor growth and patient survival [5,6,7]. Additionally, regarding these well-known metabolic alterations, the role of lipid metabolism in cancer, including EEG, has attracted growing interest in recent decades.

This research is motivated by recent advances in analytical techniques for lipid profiling [8]. The growing interest in this area stems from the increasing recognition that cancer is closely associated with the dysregulation of lipid metabolism, a factor that tumor cells can exploit to promote their growth, proliferation, and spread [9]. Moreover, many studies have indicated that reprogramming the lipid metabolism may serve as an effective strategy to overcome drug resistance in cancer cells.

Recent studies have revealed a significant association between abnormal lipid metabolism—specifically elevated triglyceride (TG) levels and decreased high-density lipoprotein cholesterol (HDL) levels—and an increased risk of developing EC. This association is of particular concern as it is associated with more aggressive forms of the disease and a higher mortality rate [10]. This highlights the importance of understanding the specific lipid changes that occur in EC and their clinical implications. For example, recent research has shown that oleic acid (OA) can enhance the activity of the PTEN/AKT/mTOR signaling pathway in EC cells. This effect could inhibit both tumor growth and the invasive properties of these cells [11]. Specifically, treatment with OA has been shown to increase *PTEN* expression and decrease the phosphorylation of PTEN, as well as *AKT* and *S6*, which are downstream targets of mTOR. Additionally, the knockdown of *PTEN* using shRNA reduced the inhibitory effect of OA, leading to decreased synthesis of TG and lipid droplet formation. This study also showed that treatment with an AKT inhibitor (ipatasertib) restored the effects of OA on cell proliferation and apoptosis [11]. Similarly, palmitic acid has also enhanced the effectiveness of certain chemotherapeutic agents against EC cells [12].

Given the diversity of FA and their various properties and cellular effects, a comprehensive analysis of the changes of these compounds and their metabolism in EC tissues is required. In this study, the relationship between FA composition and metabolism and the progression of low-grade endometrial cancer was investigated. Our aim was to evaluate the potential of these lipid alterations as biomarkers for the diagnosis of EC, predictors of disease development, and the identification of targets for possible personalized treatment strategies. Low-grade EC, which includes endometrioid G1 and G2 tumors, was selected for lipid metabolism analysis due to its highest prevalence, accounting for 75–90% of all EC cases [4].

## 2. Results

### 2.1. Anthropometric and Biochemical Characteristics of the Study Population

The biochemical and anthropometric characteristics of the study population, categorized by FIGO stages and compared to the healthy controls (HC), are shown in Table 1. The results are also visualized in the form of a heat map in Figure S1. Notably, patients with early-stage EC IA had a significantly higher BMI than HC, indicating a potential link between obesity and early-stage disease or reflecting the role of obesity in the pathogenesis of EC (Table 1). Advanced stages (II and III) of EC were associated with elevated glucose and insulin levels, pointing to a possible role of impaired glucose metabolism in the progression of the disease. This observation also suggests that compensatory mechanisms may become less effective during the advanced stages of the disease. Additionally, patients with stage III EC had elevated CRP levels compared to HC, which suggests a state of increased systemic inflammation in more advanced disease (Table 1).

While there were no significant differences observed in the lipid profile across the various stages of EC, a general trend towards dyslipidemia was noted among EC patients when all stages were considered together. Specifically, patients with EC had significantly lower HDL levels (50.8 ± 2.1 mg/dL) and higher TG levels (144.8 ± 8.3 mg/dL) compared to HC, who had HDL levels of 55.1 ± 1.7 mg/dL and TG levels of 119.0 ± 9.7 mg/dL (Table S1). This metabolic disturbance was confirmed by a significantly higher ratio of TG/HDL, TC/HDL, and LDL/HDL in all EC patients compared to HC (Table S1).

### 2.2. Changes in Total Lipid Content in the Tissue Samples

**Figure 1:**
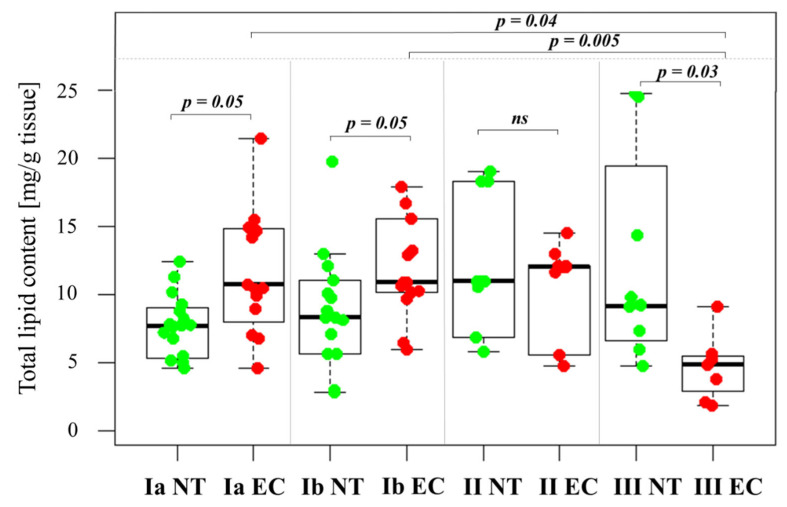
Total lipid content in normal and endometrial cancer tissue. Statistical comparisons were performed using the Kruskal–Wallis test followed by Dunn’s post-hoc test with multiple comparisons correction. *p*-values are indicated above the relevant comparisons. Green dots represent NT and red dots represent EC tissue. Note: NT—normal tissue; EC—endometrial cancer; IA, IB, II, III—stages of EC; ns—not significant.

A boxplot comparing the total lipid content (mg/g tissue) in normal control endometrial tissue (NT) and cancer tissue at different EC stages (Figure 1) shows that early-stage EC tissue (stages IA and IB) has a significantly higher lipid content than the corresponding NT. In contrast, advanced-stage III EC tissue has a lower lipid content compared to NT (Figure 1). A Kruskal-Wallis test confirmed significant differences in median lipid content between the different tumor stages and NT (chi-squared = 12.889, df = 3, *p* = 0.005), reinforcing the observed trend of decreasing lipid content with advancing cancer stage. Specifically, IA and IB together have a significantly higher total lipid content (12.619 ± 1.221 mg/g) compared to stage IIIC EC (4.668 ± 0.924 mg/g, *p* < 0.001).

Analysis of total lipid content based on their lymphovascular space invasion (LVSI) status showed lower total lipid content in LVSI-positive cases (n = 28; 6.773 ± 1.105) compared to LVSI-negative cases (n = 53; 9.741 ± 0.891). However, this difference did not reach statistical significance (*p* = 0.067). Further analysis using Spearman’s rank correlation (rho = −0.434, *p* = 0.005) and Kendall’s tau correlation (tau = −0.325, *p* = 0.008) showed a negative correlation between cancer stage and total lipid content. This correlation suggests that advancing malignancy stage is associated with a decrease in total lipid content in tumor tissue.

### 2.3. Alterations of Fatty Acid Profile in Endometrial Cancer Tissue

**Figure 2:**
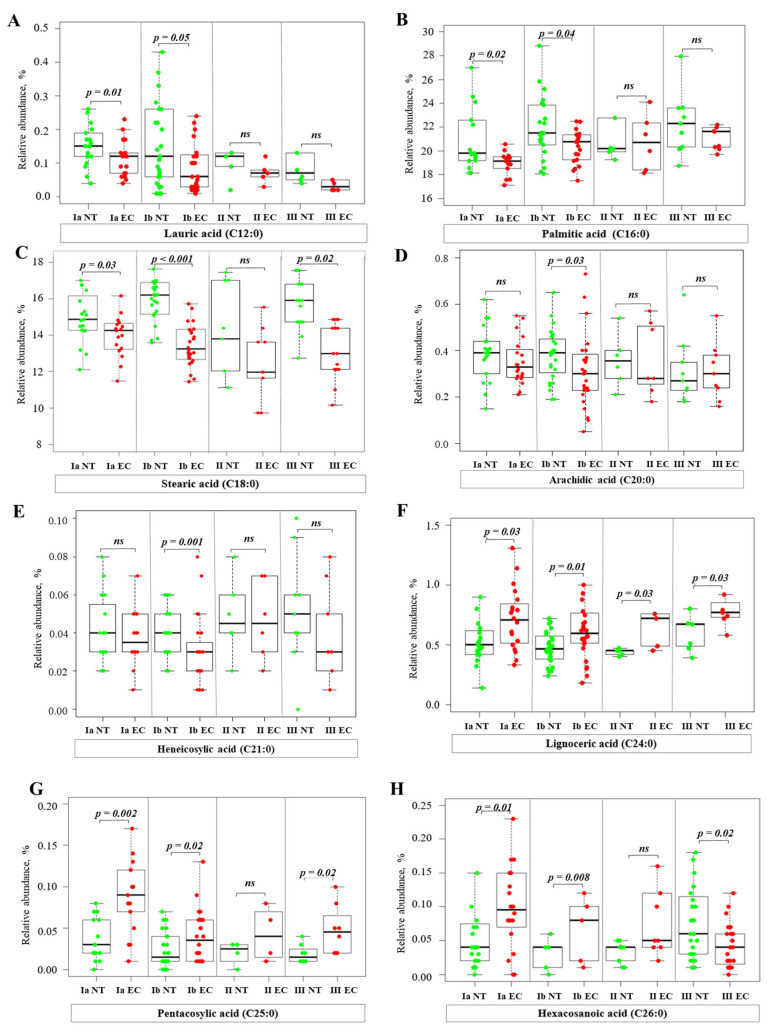
Relative abundance of SFA in normal and cancer tissues at different stages of EC. Statistical comparisons were performed using the Kruskal–Wallis test followed by Dunn’s post-hoc test with multiple comparisons correction. *p*-values are indicated above the relevant comparisons. Green dots represent NT and red dots represent EC tissue. Each subfigure shows data for a different FA: (**A**) lauric acid; (**B**) palmitic acid; (**C**) stearic acid; (**D**) arachidic acid; (**E**) heneicosylic acid; (**F**) lignoceric acid; (**G**) pentacosylic acid; (**H**) hexacosanoic acid. Note: NT—normal tissue; EC—endometrial cancer; IA, IB, II, III—stages of EC; ns—not significant.

**Figure 3:**
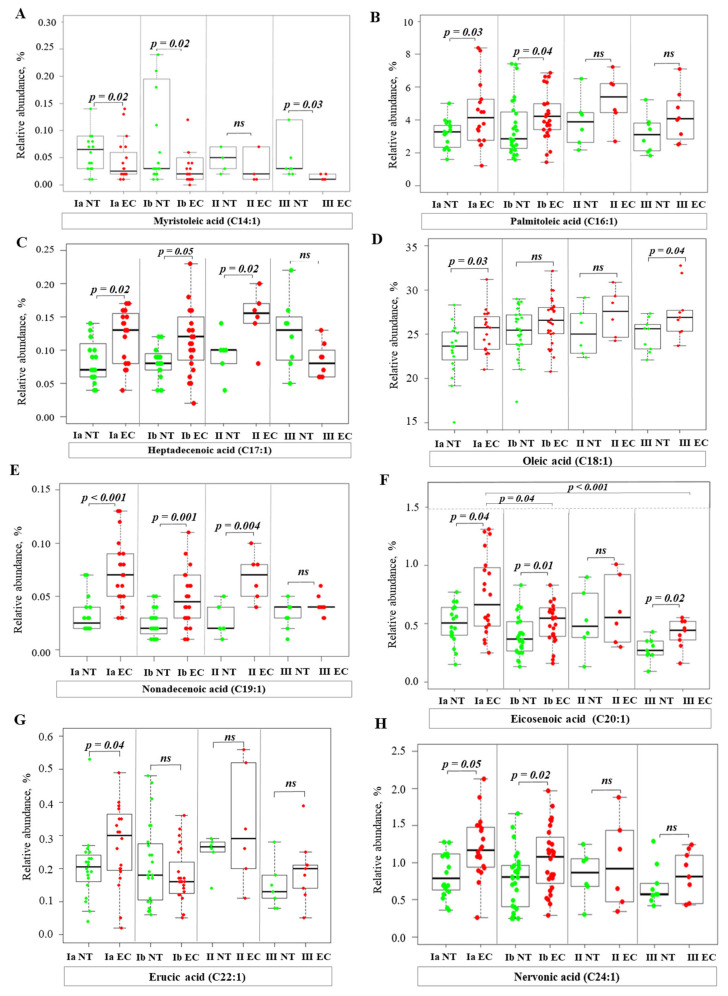
Relative abundance of MUFA in normal and cancer tissues at different stages of endometrial cancer. Statistical comparisons were performed using the Kruskal–Wallis test followed by Dunn’s post-hoc test with multiple comparisons correction. *p*-values are indicated above the relevant comparisons. Green dots represent NT and red dots represent EC tissue. Each subfigure shows data for a different MUFA: (**A**) myristoleic acid; (**B**) palmitoleic acid; (**C**) heptadecenoic acid; (**D**) oleic acid; (**E**) nonadecanoic acid; (**F**) eicosenoic acid; (**G**) erucic acid; (**H**) nervonic acid. Note: NT—normal tissue; EC—endometrial cancer; IA, IB, II, III—stages of EC; ns—not significant.

**Figure 4:**
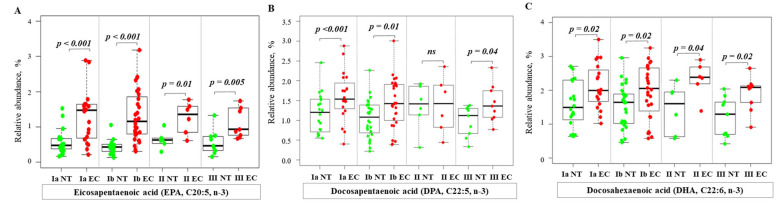
Relative abundance of n-3 PUFA in normal and cancer tissues at different stages of endometrial cancer. Statistical comparisons were performed using the Kruskal–Wallis test followed by Dunn’s post-hoc test with multiple comparisons correction. *p*-values are indicated above the relevant comparisons. Green dots represent NT and red dots represent EC tissue. Each subfigure shows data for a different n-3 PUFA: (**A**) eicosapentaenoic acid; (**B**) docosapentaenoic acid; (**C**) docosahexaenoic acid. Note: NT—normal tissue; EC—endometrial cancer; IA, IB, II, III—stages of EC; ns—not significant.

**Figure 5:**
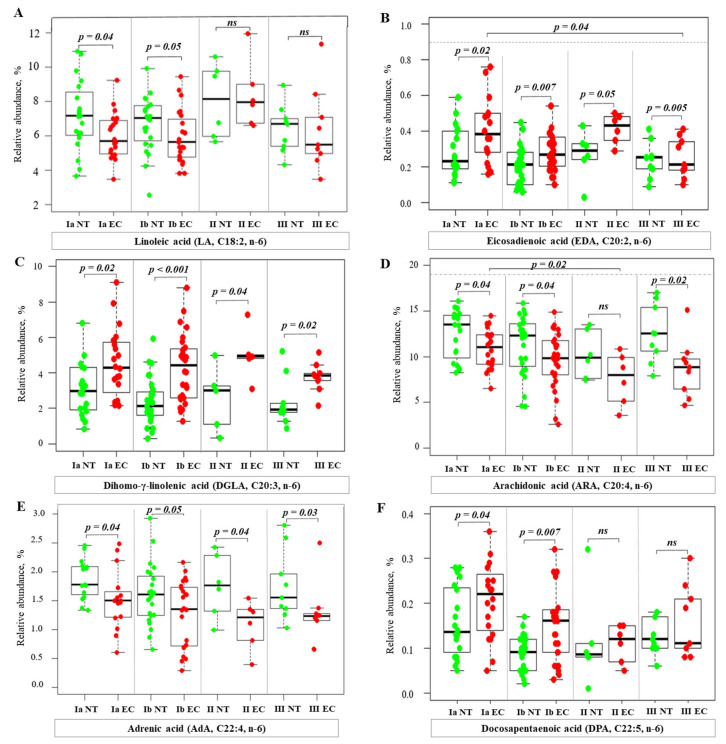
Relative abundance of n-6 PUFA in normal and cancer tissues at different stages of endometrial cancer. Statistical comparisons were performed using the Kruskal–Wallis test followed by Dunn’s post-hoc test with multiple comparisons correction. *p*-values are indicated above the relevant comparisons. Green dots represent NT and red dots represent EC tissue. Each subfigure shows data for a different n-6 PUFA: (**A**) linoleic acid; (**B**) eicosadienoic acid; (**C**) dihomo-γ-linolenic acid; (**D**) arachidonic acid; (**E**) adrenic acid; (**F**) docosapentaenoic acid. Note: NT—normal tissue; EC—endometrial cancer; IA, IB, II, III—stages of EC; ns—not significant.

**Figure 6:**
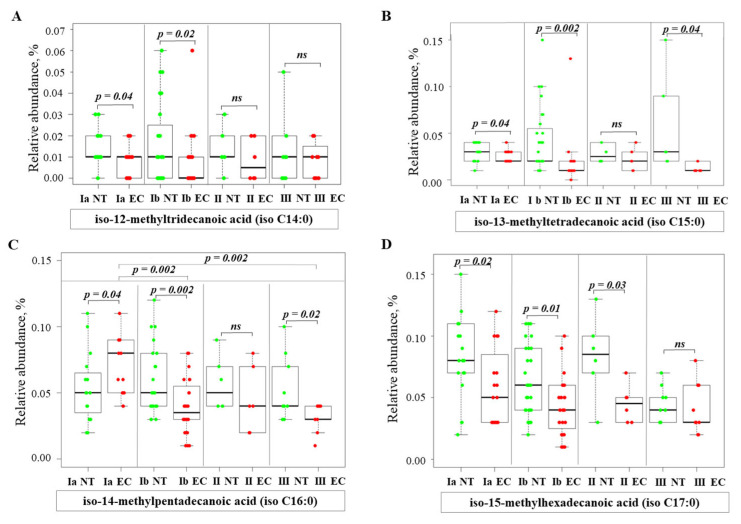
Relative abundance of iso-BCFA in normal and endometrial cancer tissues at different stages. Statistical comparisons were performed using the Kruskal–Wallis test followed by Dunn’s post-hoc test with multiple comparisons correction. *p*-values are indicated above the relevant comparisons. Green dots represent NT and red dots represent EC tissue. Each subfigure shows data for a different iso-BCFA: (**A**) iso-12-methyltridecanoic acid; (**B**) iso-13-methyltetradecanoic acid; (**C**) iso-14-methylpentadecanoic acid; (**D**) iso-15-methylhexadecanoic acid. Note: NT—normal tissue; EC—endometrial cancer; IA, IB, II, III—stages of EC; ns—not significant.

**Figure 7:**
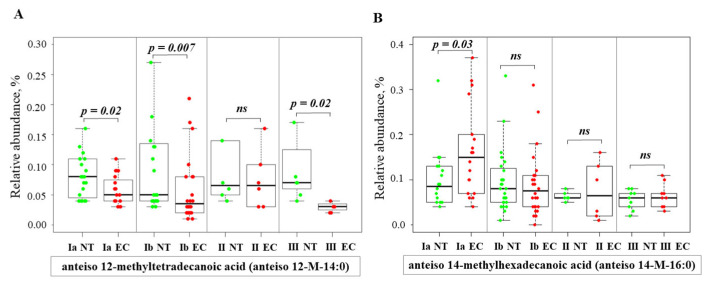
Relative abundance of anteiso-BCFA in normal and endometrial cancer tissue across different stages. Statistical comparisons were performed using the Kruskal–Wallis test followed by Dunn’s post-hoc test with multiple comparisons correction. *p*-values are indicated above the relevant comparisons. Green dots represent NT and red dots represent EC tissue. Each subfigure shows data for a different anteiso-BCFA: (**A**) anteiso 12-methyltetradecanoic acid; (**B**) anteiso 14-methylhexadecanoic acid. Note: NT—normal tissue; EC—endometrial cancer; IA, IB, II, III—stages of EC; ns—not significant.

Analysis of total FA profiles, including saturated (SFA), monounsaturated (MUFA), polyunsaturated (PUFA), and branched-chain fatty acids (BCFA), revealed significant changes in FA profiles between EC tissue and NT. The significant differences in specific FA are shown in Figure 2, Figure 3, Figure 4, Figure 5, Figure 6 and Figure 7, while the results of a full analysis can be found in Table S2.

Among SFA, the relative abundance (RA) of lauric acid (C12:0), a representative of medium-chain FA (FA with up to 12 carbons in the chain), was significantly lower in EC tissue at stages IA and IB compared to NT (Figure 2A). The RA of various long-chain FA (LCFA, 13–21 carbons) also showed significantly lower values in EC than in NT. In particular, the RA of palmitic acid (C16:0) and stearic acid (C18:0) were reduced in both stage IA and stage IB, while the RA of arachidic acid (C20:0) and heneicosylic acid (C21:0) were significantly lower only in stage IB compared to NT. In stage III, we found a lower content of C18:0 in EC tissue compared to NT (Figure 2B–E). In contrast, the very long-chain FA (VLCFA—FA with 22 or more carbons in their chains) showed an opposite trend. The RA of lignoceric acid (C24:0), pentacosanoic acid (C25:0), and hexacosanoic acid (C26:0) were significantly higher in EC tissue (Figure 2F–H). This was true for stages IA, IB, and II. A different pattern was observed in stage III EC. The RA of C24:0 and C25:0 was higher in EC, while C26:0 was higher in EC tissue compared to NT (Figure 2F–H).

The analysis of MUFA revealed different patterns. The RA of myristoleic acid (C14:1) was significantly lower in malignant tissue in stages IA, IB, and III compared to NT (Figure 3A). In contrast, the RA of other MUFA, such as palmitoleic acid (C16:1), heptadecenoic acid (C17:1), oleic acid (C18:1), nonadecenoic acid (C19:1), eicosenoic acid (C20:1), erucic acid (C22:1), and nervonic acid (C24:1), were higher in EC tissues than in NT (Figure 3B–H). Higher levels were mainly observed in stage IA; the RA of C16:1, C17:1, C19:1, C20:1, and C24:1 was also higher in stage IB (Figure 3B,C,E,F). In stage II, only the RA of C17:1 and C19:1 was significantly higher compared to NT (Figure 3C,E). In stage III, the RA of C20:1 and C18:1 was higher in EC than in NT (Figure 3F,D). In addition, the RA of C20:1 showed significant differences between stages IA and IB and between stages IA and III (Figure 3F). Both Spearman’s rank (rho = −0.32, *p* = 0.01) and Kendall’s tau (tau = −0.25, *p* = 0.01) correlation tests showed a negative correlation of C20:1 with progressive stage, despite a higher C20:1 value in EC compared to NT.

Figure 4 presents an analysis of n-3 PUFA in normal and cancerous endometrial tissue. The RA of n-3 PUFA, including eicosapentaenoic acid (EPA), docosapentaenoic acid (DPA), and docosahexaenoic acid (DHA) (Figure 4A–C), were consistently higher in cancerous tissue compared to NT at all stages of EC.

An analysis of n-6 PUFA revealed a more complex pattern (Figure 5). The RA of linoleic acid (LA), arachidonic acid (ARA), and adrenic acid (AdA) were lower in EC tissues compared to NT (Figure 5A,D,E). ARA levels progressively decreased with advancing cancer stage and showed a statistically significant negative correlation with stage (Spearman’s rho = −0.31, *p* = 0.01; Kendall’s tau = −0.24, *p* = 0.01). In contrast, RA of eicosadienoic acid (EDA), dihomo-γ-linolenic acid (DGLA), and docosapentaenoic acid (DPA) were higher in EC tissue (Figure 5B,C,F). While no significant correlation was found between the stage and RA of EDA, analyses suggest significant differences in RA levels between early-stage IA and advanced-stage III (Figure 5B).

Branched-chain FA (BCFA), which are characterized by a methyl branch in their carbon chain, exist as iso- and anteiso- isomers. The RA of iso-12-methyltridecanoic acid (iso C14:0) (Figure 6A) remained similar to NT at stage IA and showed slightly lower values at stage IB. The RA of iso-13-methyltetradecanoic acid (iso C15:0) (Figure 6B) was significantly lower in stages IA, IB, and III compared to NT. The analysis of iso-14-methylpentadecanoic acid (iso C16:0) (Figure 6C) showed higher values in stage IA, while they were lower in stages IB and III compared to NT. RA values for iso-15-methylhexadecanoic acid (iso C17:0) (Figure 6D) were lower in IA, IB, and II than in the corresponding NT samples. In general, the content of iso-BCFA in EC tissue was lower than in NT in most cases.

The RA of anteiso-12-methyltetradecanoic acid (anteiso 12-M-14:0) was lower in stages IA, IB, and III compared to NT (Figure 7A). The RA of anteiso-14-methylhexadecanoic acid (anteiso 14-M-16:0) was higher in stage IA compared to NT (Figure 7B).

### 2.4. Analysis of mRNA Levels by Real-Time PCR

**Figure 8:**
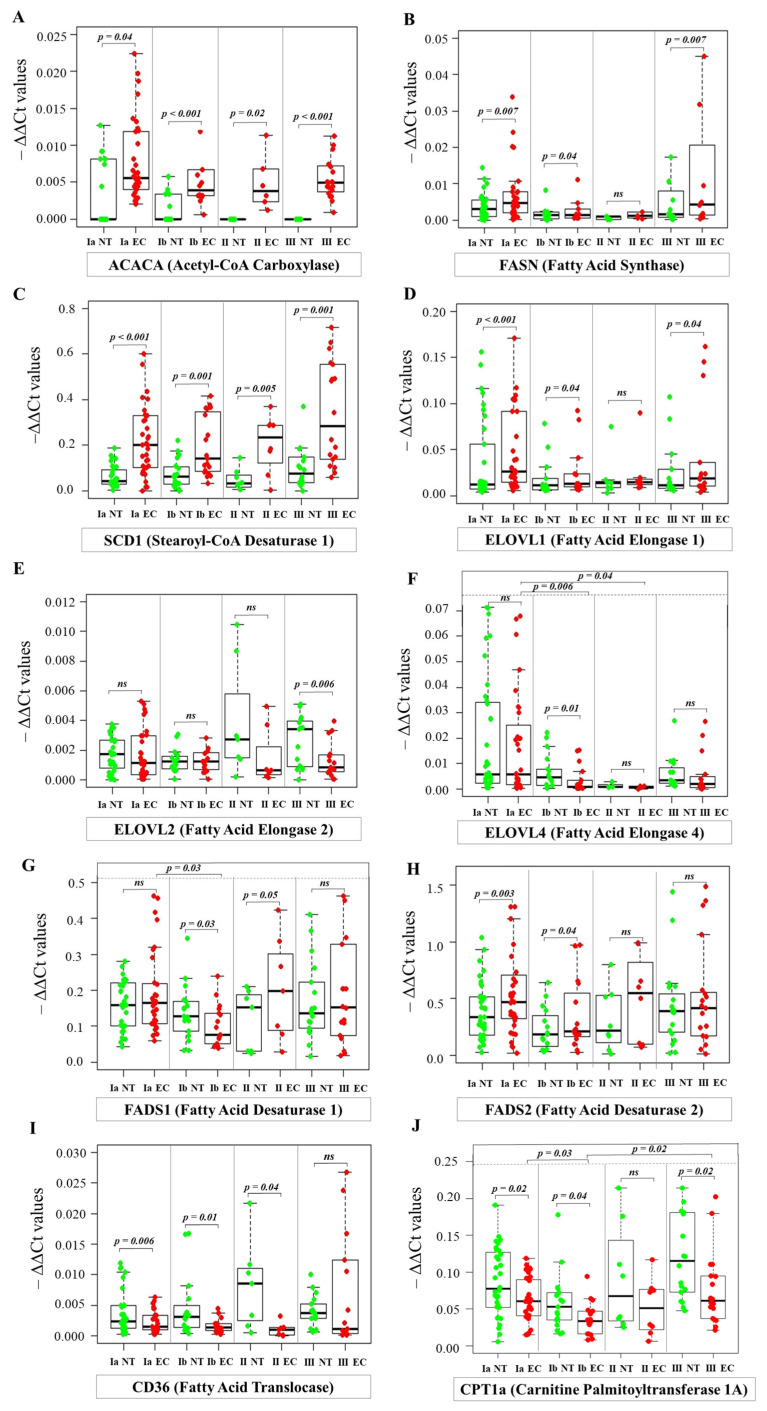
Dysregulation of lipid metabolism-related gene expression in endometrial cancer by real-time PCR. Statistical comparisons were performed using the Kruskal–Wallis test followed by Dunn’s post-hoc test with multiple comparisons correction. *p*-values are indicated above the relevant comparisons. Green dots represent NT and red dots represent EC tissue. Each subfigure displays the expression of a specific gene: (**A**) *ACACA*; (**B**) *FASN*; (**C**) *SCD1*; (**D**) *ELOVL1*; (**E**) *ELOVL2*; (**F**) *ELOVL4*; (**G**) *FADS1*; (**H**) *FADS2*; (**I**) *CD36*; (**J**) *CPT1a*. Note: NT—normal tissue; EC—endometrial cancer; IA, IB, II, III—stages of EC; ns—not significant.

To investigate the molecular mechanism of FA profile changes in cancer tissue, we also examined the expression of genes encoding proteins and enzymes responsible for FA metabolism in the cell. Figure 8 shows the dysregulated expression patterns of genes related to lipid metabolism in EC compared to NT.

Several key enzymes involved in FA synthesis show marked upregulation in EC tissue. Acetyl-CoA carboxylase (ACACA) (Figure 8A), a regulated key enzyme of FA synthesis, showed marked upregulation at all stages of EC. Specifically, *ACACA* expression was increased 3.2-fold in stage IA, 12.2-fold in stage IB, 12.5-fold in stage II, and 39058-fold in stage III compared to the control samples. While this upregulation was consistent across all stages, suggesting a persistent role of *ACACA* in EC progression, no statistically significant correlation was found between *ACACA* expression levels and tumor stage. Similarly, *FA synthase* (*FASN*) expression (Figure 8B) was increased 2.5-fold in EC tissue at stage IA, indicating significant upregulation at an early stage of the disease. Expression was also increased in stage IB, although less pronounced than in stage IA. Interestingly, this upregulation continued in advanced stage III, where a 2.9-fold increase was observed compared to NT. Of note, while the upregulation of these genes was consistent across stages, suggesting a persistent role in EC progression, no statistically significant correlation was found between their expression levels and tumor stage.

This pattern of consistent upregulation across different stages, with no significant association with tumor stage, was also observed for *stearoyl-CoA desaturase 1* (*SCD1*). *SCD1* (Figure 8F) showed a remarkably higher mRNA level in EC stages IA-III compared to NT.

The expression of members of the very long chain FA elongase (*ELOVL*) family involved in FA elongation also showed different patterns of dysregulation in EC. *ELOVL1* (Figure 8C), which was significantly upregulated in EC. Conversely, the expression of *ELOVL2* (Figure 8D) was lower in stage III EC tissues compared to NT. No significant differences in the expression of *ELOVL1* or *ELOVL2* were observed between the EC stages examined. The expression of *ELOVL4* (Figure 8E) was lower in stage IB than in NT. Importantly, our analysis revealed a statistically significant negative correlation between EC stage and *ELOVL4* expression, indicating that *ELOVL4* expression decreases with advancing tumor stage. This relationship was consistently observed in several statistical tests, including Spearman’s rank correlation (rho = −0.394, *p* = 0.0006), Kendall’s tau correlation (tau = −0.299, *p* = 0.001), and a permutation test (*p* = 0.009).

The expression of the FA desaturases *FADS1* and *FADS2* showed different patterns of dysregulation in EC. *FADS1* expression (Figure 8G) was significantly reduced in stage IB compared to NT. The decrease in *FADS1* expression during progression from stage IA to IB was statistically significant. In contrast, higher expression was observed in stage II compared to NT. In contrast to *FADS1*, *FADS2* expression (Figure 8H) was consistently higher in stages IA and IB compared to NT. No significant differences in *FADS2* expression were observed between the EC stages examined.

Our results also show changes in the expression of genes related to FA transport and oxidation. FA translocase (*CD36*) mRNA levels were significantly lower than in NT, and this down-regulation was even more pronounced in stage II, with an 8-fold decrease in *CD36* expression in EC tissues compared to NT (Figure 8I). The expression of carnitine palmitoyltransferase 1a (*CPT1a*) (Figure 8J), a key enzyme in FA oxidation, was also significantly lower in EC at stages IA, IB, and III compared to NT. Significant differences in the expression of *CPT1a* were found between stages IA and IB and between stage IB and stage III. These results indicate a possible shift in *CPT1a* expression in stage IB tumors compared to earlier and more advanced stages.

In summary, RT-PCR expression analysis showed consistent trends in all EC stages, except for *FADS1*, whose expression decreased in stage IB, while it increased in the other stages.

### 2.5. Comparison of RT-PCR and RNA-Seq Expression Profiles of Fatty Acid Metabolism Genes in Endometrial Cancer

To assess the broader relevance of our results, we compared our RT-PCR expression data for *ACACA*, *FASN*, *ELOVL1*, *ELOVL2*, *ELOVL4*, *SCD1*, *FADS1*, *FADS2*, *CD36*, and *CPT1a* at different stages of EC and NT with RNA-seq data from the UCEC dataset of TCGA. While the RNA-seq data showed a wider range of expression levels (Figure S2), the comparison of log-transformed expression ratios from RT-PCR and RNA-seq data did not show a strong correlation for the selected FA metabolic genes. This difference could be due to inherent technical differences between the two platforms or underlying biological heterogeneity within the datasets. However, a similar trend in gene expression was observed in the RNA-seq and our RT-PCR datasets. The expression patterns of these genes in the UCEC cancer stages available on UALCAN [13] are shown in Figure S3.

The prognostic significance of these genes was further tested by analyses in the GSCA database (accessed on 30 May 2024) [14,15]. Hazard ratios (HR) and associated *p*-values were calculated for overall survival (OS), progression-free survival (PFS), and disease-specific survival (DSS). Unexpectedly, the high expression of ELOVL2 was significantly associated with worse OS (HR = 2.37, *p* = 0.02) and DSS (HR = 2.73, *p* = 0.03), with a trend towards worse PFS (HR = 1.54, *p* = 0.13). Similarly, increased *ELOVL4* expression correlated with worse OS (HR = 2.20, *p =* 0.03) and DSS (HR = 2.50, *p* = 0.04), although the association with PFS was not significant (HR = 1.14, *p* = 0.64). High *ELOVL1* expression was associated with shorter PFS (HR = 1.97, *p* = 0.02). FASN expression showed non-significant associations with worse OS, PFS, and DSS (Figure S4). Furthermore, the dysregulation of FA metabolism, including altered expression of *FADS1*, *FADS2*, *ELOVL4*, and *ELOVL2*, was found to affect important cellular signaling pathways in EC, including cell cycle regulation, DNA damage response, and signaling pathways such as PI3K/AKT and RAS/MAPK (Figure S5).

## 3. Discussion

Although lipid changes in blood were observed in EC patients (Table 1), systemic blood lipid profiles are less accurate to describe the pathomechanism of lipids in cancer cells. Analysis of lipid levels directly in tumor tissue provides a more detailed understanding of lipid metabolism in cancer tissue. Interestingly, while circulating lipid levels in EC patients indicated dyslipidemia, the total lipid content in tumor tissue provided a more complex picture. In the early stages of EC (IA and IB), we observed significantly higher total lipid levels in EC tissues compared to NT (Figure 1), indicating an increase in de novo lipogenesis, which promotes rapid cell proliferation and tumor growth. This finding is consistent with the overexpression of key lipogenic genes, such as *ACACA* and *FASN* (Figure 8). In contrast, total lipid content is lower in stage III EC tissue compared to NT, suggesting that lipid reserves decrease as the cancer progresses and the tumor microenvironment changes [16]. The increased de novo lipogenesis driven by these enzymes is likely responsible for the increased metabolic demands of proliferating cancer cells. In our previous study, we also found decreased total lipid content in colorectal cancer tissue [17], indicating similar changes in lipid metabolism in different cancer types.

An analysis of individual FA levels revealed interesting trends in different EC stages. Lower levels of SFA, such as C12:0, C16:0, C18:0, C20:0, and C21:0, were observed in EC compared to NT (Figure 2A–E). While these SFA are typically produced through lipogenesis, their lower levels in EC may be attributed to their rapid consumption by other metabolic processes that are upregulated in proliferating cancer cells, such as membrane biogenesis. In contrast, EC tissue showed an increase in VLCFA, particularly C24:0, C25:0, and C26:0 (Figure 2F–H). This increase is consistent with the upregulation of *ELOVL1* (Figure 8D) at all EC stages. VLCFA are essential components of sphingolipids and other complex lipids involved in cell signaling and membrane structure [9]. The increase in VLCFA in EC may be related to changes in membrane composition and signaling pathways in cancer cells [18]. However, the observed decrease in C26:0 in stage III EC requires further investigation to understand the underlying causes and its potential impact on disease progression. Notably, a study by Zierfuss et al. [19] demonstrated that saturated VLCFA can directly affect macrophage plasticity and invasiveness. Given the established role of tumor-associated macrophages (TAM) in promoting tumor growth, angiogenesis, and metastasis, this finding suggests that the accumulation of VLCFA in EC plays a role in creating a tumor-promoting microenvironment by modulating TAM behavior [19].

The consistent upregulation of *SCD1* (Figure 8C), which is responsible for the conversion of SFA to MUFA, supports the observation of increased lipogenesis in EC. MUFA may promote tumor growth by altering membrane fluidity and enhancing pro-tumorigenic signaling pathways [20,21]. Indeed, our lipid profile analysis showed a significant increase in MUFA levels (C16:1, C17:1, C18:1, C19:1, C20:1, C22:1, and C24:1) in EC tissues compared to NT (Figure 3B–H), supporting the role of the upregulation of *SCD1* in altering the lipid landscape of EC. The increased conversion of SFA to MUFA also attenuates the intrinsic cytotoxic effects of SFA on cancer cells, thereby promoting cancer cell survival [20]. In contrast, lower levels of C14:1 were observed in EC tissues (Figure 3A). It has been shown that C14:1 exerts a pro-apoptotic effect on prostate cancer cells [22], and potentially inhibits tumor growth. An elevated serum C14:1 level has been linked to longer progression-free survival in patients with non-small cell lung cancer undergoing immunotherapy [23]. Particularly noteworthy is the negative correlation between C20:1 and tumor stage. This finding warrants further investigation to determine its potential role as a prognostic marker.

Our analysis revealed significant changes in the PUFA profile. In particular, we found higher RA of n-3 PUFA, including EPA, DHA, and DPA, in all EC stages compared to NT. Pan et al. demonstrated that DHA can suppress the PI3K/Akt signaling pathway, leading to cell cycle arrest and an anti-tumor effect in EC cells [24]. Additionally, n-3 PUFA, particularly EPA and DHA, have been shown to inhibit tumor growth in animal models of prostate, breast, and colon cancers [25].

These findings suggest a possible role of n-3 PUFA in regulating cancer cell proliferation. This observation is consistent with Mansara’s study, which showed that a lower n-6/n-3 ratio (specifically a lower ratio of ARA/EPA + DHA) resulted in lower viability and growth of breast cancer cells [26]. Higher levels of n3-PUFA in EC tissue are expected to slow the proliferation of EC cells. The RA of n-6 PUFA, such as LA, ARA, and AdA, is lower in cancer tissues. In contrast, the levels of other n-6 PUFA, such as DGLA, EDA, and DPA, are higher. This differential regulation underscores the complex role of n-6 PUFA in cancer metabolism and inflammation. Indeed, the decrease in ARA, a precursor of pro-inflammatory mediators, could be a consequence of its increased use in the production of these mediators, particularly in the context of chronic inflammation often observed in obese EC patients. Conversely, the increase in DGLA, a precursor of anti-inflammatory mediators, could be a compensatory response aimed at suppressing the inflammatory microenvironment of the tumor. However, it cannot be ruled out that the tumor microenvironment actively inhibits the conversion of DGLA into anti-inflammatory oxylipins or directly utilizes DGLA. Furthermore, the consistent decrease in the n6/n3 ratio at all stages of EC suggests that this metabolic shift occurs early in carcinogenesis and could potentially serve as a biomarker for early detection (Figure S6).

We observed the dysregulated expression of genes involved in the desaturation and elongation of PUFA, processes that are critical for maintaining the specific lipid composition required for membrane integrity and cellular signaling. The downregulation of *FADS1* (Figure 8G) in EC at stage IB is associated with a consistent upregulation of *FADS2* (Figure 8H) in early-stage IA and IB. This dysregulation may lead to changes in the levels of specific PUFA, potentially affecting membrane fluidity and signaling. Additionally, the expression patterns of *ELOVL* family members involved in the elongation of PUFA highlight the complexity of lipid metabolism dysregulation in EC. While *ELOVL1* (Figure 8D) was upregulated at all stages, *ELOVL2* (Figure 8E) showed a significant decrease in expression, particularly in stage III of EC. This stage-specific downregulation of *ELOVL2* may indicate a shift in PUFA elongation dynamics with tumor progression, potentially affecting the availability of specific longer PUFA required for membrane structure and function. Furthermore, the significant negative correlation between *ELOVL4* expression (Figure 8F) and tumor stage suggests that this elongase plays a role in early-stage EC, and its downregulation could contribute to disease progression.

We found intriguing stage-specific differences in the BCFA group. Research on BCFA in cancer is still ongoing, but some studies have suggested that they may have both pro-inflammatory and anti-inflammatory effects, influencing various cellular processes such as apoptosis, cell cycle regulation, and angiogenesis [27]. Wongtangtintharn et al. described the antitumor activity of BCFA in human breast cancer cells, focusing on their effect on FA biosynthesis [28]. Remarkably, iso-C16:0 was significantly elevated in stage IA EC compared to the control samples, suggesting that it could serve as a potential early-stage biomarker. This observation emphasizes the need for further investigation of the complex interplay between BCFA and the development of EC, which may lead to new diagnostic and therapeutic strategies.

Our results also suggest alterations in FA transport and oxidation, highlighting metabolic reprogramming in EC. The consistent down-regulation of *CD36* (Figure 8I), a major FA transporter, in all EC stages—especially in stage II—suggests a possible impairment of FA uptake. This decreased FA uptake may result from an increase in de novo lipogenesis, as cancer cells favor endogenous FA synthesis over exogenous uptake. Similarly, the downregulation of *CPT1a* (Figure 8J), a rate-limiting enzyme in FA oxidation, at the different EC stages, especially stages IA, IB, and III, suggests a possible shift away from FA oxidation as an energy source. This metabolic shift could be related to the increased reliance on glycolysis, which is a common feature of cancer cells, even when oxygen is available.

Our study examines gene expression and FA profiles at different stages of EC. However, it does not fully elucidate the exact molecular mechanisms by which these changes promote tumor development and progression. Future research should clarify these mechanisms. In particular, in vitro experiments with EC cell lines could investigate the effects of modulating key enzymes involved in FA metabolism on cancer cell proliferation, migration, and invasion. For instance, the treatment of EC cells with inhibitors of *ACACA, ELOVL1*, or *FASN* would directly test the hypothesis that increased de novo lipogenesis promotes tumor growth. Conversely, the stimulation of FA oxidation by *CPT1a* activators could help determine whether restoration of this pathway inhibits EC progression. Such in vitro results could then be validated in vivo using preclinical animal models of EC.

As a limitation of our study, we have to mention the limited number of samples in more advanced EC stages, such as stage II, which reflects clinical and practical limitations, as early-stage EC is more commonly diagnosed and surgically treated. We acknowledge that the smaller sample sizes in these subgroups may limit the statistical power of stage-specific comparisons.

## 4. Materials and Methods

### 4.1. Patients

The study included 83 EC patients who underwent surgery at the Department of Obstetrics and Gynaecology of the University Medical Centre, affiliated with the Medical University of Gdansk (MUG). The inclusion criteria were defined as follows: patients with a primarily diagnosed G1 or G2 endometrioid EC at all stages, according to the FIGO 2009 classification [29]. Additionally, all participants were scheduled for radical treatment of hysterectomy (removal of the uterus). Finally, the presence of macroscopically visible cancerous tissue within the endometrium with corresponding normal endometrial tissue had to be identifiable. The exclusion criteria were as follows: age under 18 years, previous fertility-preserving treatment with local endometrial hormonal therapy, neoadjuvant treatment for endometrial cancer, and previously received chemotherapy for any other cancer within the past 2 years. The study was conducted in accordance with the Declaration of Helsinki of the World Medical Association and the approval of the MUG Ethics Committee (Decision No. NKBBN/9/2021). All patients who participated in the study were informed about the study and signed a written informed consent form. Preoperative blood samples were taken from each EC patient and routinely analyzed biochemically in the Central Clinical Laboratory of the MUG using standard clinical laboratory techniques. Subjects in the control group were selected from a cohort of 58 healthy women, all of whom had undergone an endometrial biopsy that confirmed normal endometrial tissue and met the established exclusion criteria for the study. Their serum biochemical values were then analyzed. Subjects in the control group were selected from a cohort of 58 healthy women, all of whom had undergone an endometrial biopsy that confirmed normal endometrial tissue and met the established exclusion criteria for the study. Anthropometric measurements were also performed.

### 4.2. Tissue Collection

During the surgical procedure, immediately after removing the uterus, an incision was made in the uterine wall to access the uterine cavity. Fragments of both normal endometrial tissue (NT) and cancerous tissue, measuring up to 8 mm, were collected. The NT was obtained from the region without macroscopic lesions. A histological examination conducted after surgery confirmed the diagnosis of low-grade endometrioid EC. The tissue samples from patients with EC were diagnosed in the following FIGO stages: IA with cancer confined to the endometrium or with superficial infiltration of the myometrium (n = 36), IB with deep cancer infiltration of the myometrium accounting for more than 50% of the wall thickness (n = 19), II with stromal invasion of the cervix (n = 9), and III with cancer infiltration of regional organs (n = 19), including IIIA uterine serosa (n = 5), IIIB adnexa (n = 2), and IIIC parametria or regional lymph nodes (n = 12). All tissue samples were immediately frozen in liquid nitrogen and stored at −80 °C until analysis. NT from the same patients was used as a control for the tissue analyses. This paired design allowed a direct comparison between NT and cancer tissue within the same individual and thus minimized the influence of inter-individual variability.

### 4.3. Fatty Acids Analysis

FA profiles from the tissue sample were determined by gas chromatography and mass spectrometry (GC-MS). Briefly, total lipids were extracted from the tissue samples using a chloroform-methanol mixture (2:1, *v*/*v*), following the method described by Folch et al. [30]. After drying, the extracted lipids were then dried under nitrogen and hydrolyzed with 0.5 M KOH at 90 °C for 3 h. After incubation, the mixtures were acidified with 6 M HCl, and 1 mL of water was added. The non-esterified FA were extracted three times using 1 mL n-hexane, and the organic phase was evaporated under nitrogen. To convert the extracted FA into fatty acid methyl esters (FAME), they were derivatized with a 10% boron trifluoride in a methanol solution at 55 °C for 1.5 h. Afterward, 1 mL of water was added, and the FAMEs were extracted with n-hexane (3 × 1 mL); the extracts were then dried under nitrogen and stored at −20 °C until analysis.

FAME were analyzed using a GC-EI-MS QP-2020 NX (Shimadzu, Kyoto, Japan) with chromatographic separation on a Zebron ZB-5MSi capillary column, 30 m × 0.25 mm i.d. × 0.25 μm film thickness (Phenomenex, Torrance, CA, USA). A 1 μL sample was injected in split mode using dichloromethane as the solvent. The column temperature was set in a range of 60 °C to 300 °C at a rate of 4 °C/min using helium as the carrier gas at a column head pressure of 60 kPa. The temperature of the injection, ion source, and transfer line was 300 °C. The ionization of FAME was performed by electron ionization at 70 eV. Additionally, 19-methylarachidic acid was used as an internal standard. Full scan mode with a mass scan range of *m*/*z* 45 to 700 was used. Accurate identification of the FA profile was possible by reference to the FAME mixture standards (Larodan, MI, USA, and Merck, Darmstadt, Germany).

### 4.4. Real-Time PCR Analysis of mRNA Levels

Total RNA was extracted from frozen tissues using the RNeasy Plus Universal MiniKit (Qiagen, Hilden, Germany, 73404) according to the manufacturer’s protocol. The quality and quantity of extracted RNA were determined using a NanoDrop spectrophotometer (Thermo Fisher Scientific, Waltham, MA, USA) and an ExperionTM automated gel electrophoresis system (Bio-Rad Laboratories, Hercules, CA, USA). Total RNA was then reverse transcribed into cDNA using a RevertAid First Strand cDNA Synthesis Kit (Thermo Fisher Scientific, Waltham, MA, USA, K1622) and stored at −20 °C until analysis. Quantitative real-time PCR was performed using a CFX Connect Real-Time PCR Detection System (Bio-Rad) and a SensiFAST SYBR No-ROX Kit (Meridian Bioscience, Cincinnati, OH, USA, BIO-98020). The cyclophilin A gene was used as a reference gene. The primers for the PCR reaction for the ACACA, FASN, ELOVL1, ELOVL2, ELOVL4, SCD1, FADS1, FADS2, CD36, and CPT1a genes were synthesized by Genomed S.A. (Warsaw, Poland), and their sequences are presented in Table S3. The data were analyzed using the relative 2^−△△Ct^ quantification method.

### 4.5. Statistical Analysis

Statistical analyses were conducted with SigmaPlot (Systat Software Inc., San Jose, CA, USA; version 14.5) and R (R Foundation for Statistical Computing, Vienna, Austria; version 4.2.2). To evaluate the normality of the data distribution, Shapiro-Wilk testing was implemented to assess the normality of the data distribution. Differences between groups were analyzed using the Kruskal-Wallis test for non-normally distributed data, followed by Dunn’s post-hoc test with *p*-values adjusted for multiple testing using Holm or Bonferroni correction methods. For normally distributed data, one-way ANOVA was employed, followed by either Tukey’s or the Holm-Sidak post-hoc tests with the abovementioned corrections for multiple comparisons. Correlations between variables were assessed using Spearman’s rank correlation and Kendall’s tau correlation test when parametric assumptions were not met. A *p*-value < 0.05 was considered statistically significant in all statistical tests performed. RNA-seq data for the Uterine Corpus Endometrial Carcinoma (UCEC) cohort from The Cancer Genome Atlas (TCGA) project [31] were downloaded from the Genomic Data Commons portal [32]. Gene expression analysis based on cancer stage was performed using UALCAN (The College of Alabama at Birmingham Cancer Data Analysis Portal), a web portal for analyzing TCGA data [13]. To assess the correlation between RNA-seq and our RT-qPCR expression data, RNA-seq reads were log2-transformed, with a pseudocount of +1 added to stabilize the variance. RT-qPCR data were normalized using the scaling function in R, with the data centered and scaled to a standard deviation of 1. Pearson, Kendall, and Spearman correlation coefficients were then calculated for each gene and visualized using the ggplot2 package. Gene Set Cancer Analysis (GSCA) was used to perform survival analyses across the investigated gene sets and pathways [14,15]. All post-hoc comparisons included corrections for multiple testing to reduce the risk of Type I errors.

## 5. Conclusions

Our comprehensive analysis of EC tissue revealed significant reprogramming of FA metabolism, highlighting its critical role in cancer progression. In particular, stage-dependent changes were found, with significant shifts observed in C20:1, EDA, ARA, and iso-C16:0. These findings emphasize the dynamic and stage-specific nature of FA metabolism in EC, suggesting that metabolic alterations are not merely by-products of malignancy but actively contribute to tumor growth and progression.

The high levels of VLCFA and MUFA, as well as the upregulation of key lipogenic enzymes such as *ACACA*, *FASN*, *SCD1*, and *ELOVL1*, suggest that de novo lipogenesis is a central metabolic pathway in the development of EC. This shift in lipogenesis is supported by the observed decrease in FA oxidation and FA transport, as evidenced by the downregulation of *CPT1a* and *CD36*, respectively. The complex interplay between anti-inflammatory and pro-inflammatory PUFA reveals opposing trends in n-3 and n-6 PUFA, reflecting a balance that may influence the tumor microenvironment and immunomodulation. These metabolic adaptations not only meet the energy and biosynthetic requirements of rapidly proliferating cancer cells but also highlight critical pathways of lipid metabolism in EC that could be targeted for therapeutic intervention.

## Figures and Tables

**Table 1:** Biochemical and anthropometric characteristics of patients with endometrial cancer.

	Characteristic	Control (HC)	Stage IA	Stage IB	Stage II	Stage III	*p*-Value
Sample size	n	58	36	19	9	19	-
Demographic characteristics	Age (years)	58.3 ± 1.2	59.0 ± 2.1	64.6 ± 1.8	69.7 ± 4.0	65.7 ± 1.4	1a vs. II *p* = 0.04
Body composition	BMI (kg/m^2^)	26.7 ± 0.6	33.4 ± 1.4	31.6 ± 1.7	29.5 ± 2.5	33.1 ± 1.9	HC vs. III *p* = 0.02 HC vs. IA *p* = 0.002
Glycemic analysis	HbA1c (%)	5.7 ± 0.09	5.9 ± 0.2	5.7 ± 0.3	6.6 ± 0.5	6.5 ± 0.3	ns
	Glucose (mg/dL)	89.6 ± 2.7	98.8 ± 4.3	101.6 ± 4.7	130.2 ± 17.7	128.0 ± 11.3	1a vs. III *p* = 0.02 HC vs. III *p* < 0.001
	Insulin (µU/mL)	6.5 ± 0.7	11.1 ± 1.6	9.7 ± 1.9	16.6 ± 3.6	10.1 ± 1.8	HC vs. II *p* = 0.02
	HOMA-IR	1.2 ± 0.09	3.1 ± 0.6	2.6 ± 0.6	5.4 ±1.8	3.1 ± 0.6	HC vs. II *p* = 0.03 HC vs. III *p* = 0.01
Inflammatory marker	CRP (mg/dL)	2.2 ± 0.3	7.5 ± 3.2	3.3 ± 0.8	3.3 ± 0.6	8.0 ± 2.4	HC vs. III *p* = 0.04
Tumor markers	AFP (ng/mL)	2.5 ± 0.2	4.2 ± 1.0	3.9 ± 0.7	3.0 ± 1.1	2.4 ± 0.4	HC vs. IA *p* = 0.009 HC vs. IB *p* = 0.050
	β-HCG (IU/L)	2.0 ± 0.2	1.7 ± 0.3	2.9 ± 0.4	2.7 ± 0.9	3.1 ± 0.6	HC vs. IB *p* = 0.028 HC vs. III *p* = 0.034 1a vs. IB *p*= 0.04 1a vs. III *p* = 0.012
	CEA (ng/mL)	1.6 ± 0.1	1.4 ± 0.2	2.3 ± 0.6	1.9 ± 0.3	2.8 ± 0.5	HC vs. III *p* = 0.009 1a vs. III *p* = 0.046
	Ca19 -9 (U/mL)	5.9 ± 0.8	17.8 ± 12.6	102.3 ± 49.9	169.5 ± 146.7	113.6 ± 70.7	HC vs. IB *p* = 0.007 HC vs. II *p* = 0.012 HC vs. III *p* = 0.002
	Ca125 (U/mL)	14.2 ± 0.8	44.8 ± 27.2	47.3 ± 17.6	30.9 ± 11.8	45.9 ± 14.3	HC vs. IA *p* = 0.050 HC vs. IB *p* = 0.015 HC vs. II *p* = 0.014 HC vs. III *p* = 0.004
Lipid profiles	TC (mg/dL)	192.1 ± 6.7	196.5 ± 7.8	213.6 ± 14.8	199.5 ± 18.2	182.5 ± 10.6	ns
	HDL (mg/dL)	55.1 ± 1.7	49.0 ± 2.7	56.8 ± 3.9	47.5 ± 4.5	49.9 ± 6.9	ns
	LDL (mg/dL)	118.9 ± 6.1	132.6 ± 8.2	138.4 ± 14.0	132.2 ± 18.6	114.1 ± 10.7	ns
	TG (mg/dL)	119.0 ± 9.7	140.8 ± 12.2	130.9 ± 11.4	150.0 ± 27.9	167.8 ± 23.7	ns
	TG/HDL ratio	2.4 ± 0.2	3.4 ± 0.5	2.5 ± 0.3	3.4 ± 2.1	4.2 ± 0.9	HC vs. III *p* = 0.05
	TC/HDL ratio	3.6 ± 0.1	4.3 ± 0.3	3.9 ± 0.3	4.3 ± 0.4	4.0 ± 0.3	ns
	LDL/HDL ratio	2.2 ± 0.1	3.0 ± 0.2	2.5 ± 0.3	2.8 ± 0.4	2.5 ± 0.3	ns
Medication use (yes; %)	diabetes medications	25%	22%	21%	33%	53%	-
	cholesterol-lowering medication	36%	25%	16%	33%	37%	-
Hormonal status (yes; %)	Postmenopausal	91%	75%	84%	89%	100%	-

Note: Values are the mean ± SEM. Abbreviations: HC—healthy control, BMI—body mass index; HbA1c—glycosylated haemoglobin A1c; HOMA-IR—homeostatic model assessment for insulin resistance; CRP—C-reactive protein, AFP—alpha-fetoprotein; β-HCG—β-Human chorionic gonadotropin; CEA—carcinoembryonic antigen; Ca19-9—carbohydrate antigen 19-9; Ca125—cancer antigen 125; TC—total cholesterol; HDL- high-density lipoprotein cholesterol; LDL—low-density lipoprotein cholesterol; TG—triglycerides; kg/m^2^—kilograms per square meter; mg/dL—milligrams per deciliter; µU/mL—micro units per milliliter; ng/mL—nanograms per milliliter; IU/L—international units per liter; U/mL—units per milliliter.

## Data Availability

The RNA-seq data used for comparison are publicly available through TCGA via the GDC data portal (https://portal.gdc.cancer.gov/; accessed on 30 May 2024). The UALCAN portal (http://ualcan.path.uab.edu/; accessed on 30 May 2024) and GSCA (https://guolab.wchscu.cn/GSCA/#/; accessed on 30 May 2024) were used to access and analyze TCGA data. The raw data from our gene expression and fatty acid profiling experiments are available upon reasonable request from the corresponding author after signing a data access agreement to ensure ethical considerations and data privacy.

## Supplementary Materials

The following supporting information can be downloaded at: https://www.mdpi.com/article/10.3390/ijms26073322/s1.

## Abbreviations

The following abbreviations are used in this manuscript:

B	BCFA	monomethyl branched-chain fatty acids
D	DSS	disease-specific survival
E	EC	endometrial cancer
F	FA	fatty acid
	FAME	fatty acid methyl esters
	FIGO	The International Federation of Gynecology and Obstetrics
G	GSCA	Gene Set Cancer Analysis
H	HC	healthy controls
	HDL	high-density lipoprotein cholesterol
	HOMA-IR	homeostatic model assessment for insulin resistance
	HR	hazard ratios
L	LCFA	long-chain fatty acids
	LDL	low density lipoprotein cholesterol
	LVSI	lymphovascular space invasion
M	MUFA	monounsaturated fatty acid
	MUG	Medical University of Gdansk
N	NT	normal endometrial tissue
O	OA	oleic acid
	OS	overall survival
P	PFS	progression-free survival
	PUFA	polyunsaturated fatty acid
R	RA	relative abundance
S	SFA	saturated fatty acids
T	TCGA	the cancer genome atlas
	TG	triglyceride
U	UALCAN	The University of Alabama at Birmingham Cancer Data Analysis Portal
	UCEC	uterine corpus endometrial carcinoma
V	VLCFA	very long chain fatty acids
