# Supplementary material for: Endometrial Cancer Is Associated with Altered Metabolism and Composition of Fatty Acids
Source: Int J Mol Sci. 2025 Apr 2;26(7):3322. doi: 10.3390/ijms26073322 (PMC11989365; doi:10.3390/ijms26073322)
Supplement: Supplementary file 1 [file ijms-26-03322-s001.zip › ijms-3521934-supplementary.pdf]

Table S1. Comparison of lipid profiles between healthy controls and endometrial cancer patients

	Healthy control (n = 58)	EC patients (n = 83)	*p-value*
**TC**	192.1 ± 6.7	197.9 ± 5.7	*ns*
**HDL**	55.1 ± 1.7	50.8 ± 2.1	*0.020*
**LDL**	118.9 ± 6.1	130.2 ± 5.7	*ns*
**TG**	119.0 ± 9.7	144.8 ± 8.3	*0.003*
**TG/HDL ratio**	2.4 ±0.2	3.4 ± 0.3	*0.002*
**TC/HDL ratio**	3.6 ±0.1	4.2 ± 0.2	*0.005*
**LDL/HDL ratio**	2.2 ± 0.1	2.8 ± 0.1	*0.010*

Note: Values are the mean ± SEM. EC patients (stages Ia, Ib, II and III together). Abbreviations: TC - total cholesterol; HDL- high-density lipoprotein cholesterol; LDL- low-density lipoprotein cholesterol; TG – triglycerides.

Table S2. **Fatty acids profile (relative abundance) in normal tissue (NT) and endometrial cancer tissue (EC)**

	Endometrial cancer								*p-value*			
	Ia		Ib		II		III		Ia	Ib	II	III
	n=36		n=19		n=9		n=19					
	NT	EC	NT	EC	NT	EC	NT	EC	NT EC	NT EC	NT EC	NT EC
10:0	0.006± 0.003	0.011± 0.004	0.010± 0.005	0.010± 0.002	traces	traces	ND	ND	0.368	0.463	-	-
12:0	0.152±0.015	0.113±0.013	0.199±0.041	0.107±0.023	0.165±0.054	0.112±0.041	0.154±0.080	0.067±0.027	**0.015**	**0.046**	0.310	0.373
14:0	1.284± 0.110	1.273± 0.091	1.431± 0.151	1.265± 0.110	1.488± 0.289	1.500± 0.200	1.357± 0.317	1.231± 0.160	0.766	0.941	0.974	0.791
16:0	21.443+0.925	19.122±0.557	21.944±0.588	20.497±0.364	21.613±1.256	20.730±0.948	22.338±0.889	21.719±0.777	**0.020**	**0.043**	0.587	0.453
18:0	15.641±0.679	14.005±0.385	16.557±0.685	13.601±0.359	13.997±1.113	12.420±0.912	16.064±0.434	15.222±0.660	**0.031**	**<0.001**	0.299	0.302
20:0	0.384± 0.036	0.352± 0.022	0.380± 0.021	0.328± 0.035	0.357± 0.046	0.343± 0.066	0.313± 0.048	0.312± 0.041	0.348	**0.033**	0.872	0.986
22:0	0.426± 0.032	0.468± 0.036	0.441± 0.027	0.422± 0.037	0.413± 0.036	0.445± 0.099	0.433± 0.031	0.442± 0.046	0.288	0.346	0.770	0.875
24:0	0.555±0.057	0.711±0.058	0.486± 0.030	0.701± 0.080	0.438± 0.012	0.722± 0.132	0.600± 0.055	0.799± 0.059	**0.033**	**0.010**	**0.026**	**0.030**
26:0	0.063± 0.017	0.107±0.016	0.041±0.006	0.096±0.020	0.038± 0.010	0.066± 0.022	0.031±0.005	0.092±0.025	**0.014**	**0.008**	0.413	**0.022**
28:0	0.003± 0.002	0.016± 0.002	0.009± 0.002	0.011±0.002	0.010±0.000	0.013± 0.003	0.007± 0.002	0.014± 0.004	**0.001**	0.514	0.374	0.181
Total ECFA	4.014± 0.561	3.640± 0.494	4.183± 0.495	3.691± 0.433	4.804±1.148	4.434± 1.054	4.874±0.947	4.642± 0.903	0.700	0.699	0.942	0.897
11:0	traces	traces	traces	traces	NT	NT	NT	NT	-	-	-	-
13:0	0.023± 0.003	0.016± 0.002	0.036± 0.007	0.019± 0.005	0.058± 0.034	0.028± 0.010	0.038± 0.020	0.020± 0.007	0.095	0.054	0.589	0.746
15:0	0.573±	0.528±	0.63±	0.54±	0.71±	0.70±	0.62±	0.54±	0.989	0.481	0.699	0.860

	0.067	0.040	0.069	0.052	0.17	0.16	0.15	0.08				
17:0	0.433± 0.039	0.516± 0.038	0.384± 0.028	0.431± 0.037	0.395± 0.063	0.533± 0.101	0.422± 0.043	0.400± 0.051	0.138	0.313	0.273	0.742
19:0	0.058± 0.016	0.047± 0.004	0.048± 0.004	0.044± 0.003	0.050± 0.006	0.063± 0.011	0.063± 0.013	0.044± 0.007	0.858	0.570	0.323	0.265
21:0	0.041± 0.004	0.038± 0.003	0.044± 0.004	0.030± 0.003	0.048± 0.008	0.047± 0.008	0.052± 0.010	0.037± 0.008	0.543	**0.001**	0.891	0.252
23:0	0.092± 0.011	0.106± 0.010	0.096± 0.006	0.108± 0.009	0.108± 0.018	0.150± 0.035	0.109± 0.008	0.117±0.01 4	0.197	0.406	0.310	0.634
25:0	0.046± 0.013	0.089± 0.010	0.026± 0.005	0.050± 0.010	0.014± 0.008	0.036± 0.014	0.019± 0.004	0.048± 0.010	**0.002**	**0.025**	0.219	**0.022**
Total OCFA	0.184± 0.021	0.194± 0.020	0.180± 0.019	0.177± 0.017	0.202± 0.046	0.227± 0.049	0.192± 0.035	0.176± 0.029	0.331	0.717	0.636	0.899
Total SFA	2.353±0.3 35	2.146± 0.295	3.272±0.39 0	2.896± 0.344	3.529±0.86 4	3.368±0.83 5	3.664±0.83 3	3.562±0.82 2	0.375	0.787	0.474	0.925
iso 12–M–13:0	0.014±0.0 02	0.008± 0.002	0.018± 0.004	0.007± 0.003	0.013± 0.004	0.008±0.00 4	0.020±0.00 9	0.009± 0.003	**0.041**	**0.021**	0.411	0.614
iso 13–M–14:0	0.035± 0.005	0.025± 0.001	0.042±0.00 7	0.022± 0.005	0.035± 0.010	0.022± 0.005	0.057± 0.022	0.018± 0.007	**0.049**	**0.003**	0.310	**0.037**
iso 14–M–15:0	0.081± 0.010	0.054± 0.007	0.065± 0.007	0.042± 0.005	0.057± 0.008	0.045± 0.010	0.053± 0.008	0.031± 0.004	**0.044**	**0.002**	0.400	**0.024**
iso 15–M–16:0	0.091±0.0 10	0.059± 0.007	0.068± 0.007	0.046± 0.006	0.083± 0.014	0.045± 0.006	0.044± 0.005	0.041±0.00 7	**0.018**	**0.012**	**0.028**	0.698
iso 20–	0.009± 0.002	0.004± 0.001	0.010± 0.002	0.005± 0.001	0.008± 0.003	0.003± 0.002	0.011± 0.003	0.010±0.00 2	**0.037**	**0.045**	0.209	0.948

M–21:0												
iso 22–M–23:0	0.016±0.006	0.004±0.001	0.014±0.002	0.005±0.001	0.012±0.006	ND	0.019±0.004	0.013±0.002	**0.049**	**0.010**	-	0.531
Total iso BCFA	0.036±0.004	0.028±0.003	0.037±0.003	0.022±0.002	0.035±0.006	0.021±0.004	0.033±0.004	0.021±0.002	0.051	**<0.001**	0.082	0.052
anteiso 12–M–14:0	0.094±0.016	0.057±0.005	0.121±0.026	0.072±0.020	0.107±0.038	0.075±0.020	0.101±0.027	0.050±0.022	**0.024**	**0.007**	0.699	**0.021**
anteiso 14–M–16:0	0.099±0.016	0.163±0.024	0.095±0.013	0.101±0.019	0.063±0.004	0.075±0.026	0.056±0.007	0.064±0.009	**0.025**	0.605	1.000	0.454
Total anteiso BCFA	0.096±0.011	0.107±0.014	0.108±0.014	0.087±0.014	0.085±0.019	0.075±0.016	0.076±0.013	0.058±0.011	0.851	**0.028**	0.684	0.149
14:1	0.080±0.015	0.043±0.009	0.250±0.081	0.099±0.035	0.106±0.064	0.098±0.071	0.258±0.162	0.098±0.056	**0.023**	**0.017**	0.886	**0.031**
16:1	3.145±0.196	4.414±0.469	3.548±1.319	4.449±1.356	3.910±0.635	5.225±0.663	3.131±0.408	4.216±0.557	**0.029**	**0.042**	0.850	0.868
17:1	0.094±0.012	0.124±0.012	0.101±0.015	0.120±0.012	0.093±0.013	0.150±0.016	0.114±0.019	0.084±0.009	**0.025**	**0.047**	**0.023**	0.165
18:1	23.247±0.689	25.309±0.555	25.304±0.509	26.475±0.488	25.285±1.112	27.378±1.077	24.881±0.625	27.516±1.004	**0.026**	0.103	0.206	**0.041**
19:1	0.037±0.007	0.072±0.007	0.028±0.004	0.057±0.009	0.027±0.006	0.068±0.009	0.033±0.004	0.041±0.003	**<0.001**	**0.001**	**0.004**	0.148
20:1	0.541±	0.742±0.076	0.418±0.044	0.565±0.053	0.520±0.113	0.612±0.121	0.278±0.032	0.413±0.041	**0.045**	**0.012**	0.592	**0.020**

	0.060											
22:1	0.204± 0.022	0.294± 0.039	0.176± 0.017	0.213± 0.026	0.248± 0.022	0.328± 0.073	0.194±0.031	0.196±0.059	**0.040**	0.266	0.589	0.330
24:1	0.930± 0.102	1.209± 0.093	0.772± 0.072	1.043± 0.082	0.835± 0.139	0.993± 0.248	0.693± 0.092	0.813± 0.110	**0.049**	**0.016**	0.589	0.414
Total MUFA	3.414± 0.600	3.885± 0.651	4.066± 0.567	4.066± 0.593	3.925± 1.203	4.318± 1.299	3.811± 0.965	4.178± 1.067	0.292	0.627	0.573	0.943
ALA	0.094± 0.011	0.067± 0.007	0.064± 0.006	0.065± 0.008	0.078± 0.017	0.097± 0.023	0.058± 0.010	0.052± 0.011	0.050	0.514	0.394	0.221
ETA	0.049± 0.005	0.058± 0.010	0.038± 0.004	0.041± 0.004	0.022± 0.004	0.057± 0.018	0.033±0.006	0.042± 0.012	0.731	0.485	0.240	0.714
EPA	0.570± 0.078	1.379± 0.192	0.533± 0.077	1.310±0.138	0.630± 0.099	1.253±0.185	0.562±0.118	1.397±0.342	**<0.001**	**<0.001**	**0.014**	**0.005**
DPA	1.179± 0.113	1.607±0.137	1.043±0.090	1.438±0.118	1.343± 0.241	1.392±0.294	0.974± 0.129	1.429± 0.160	**<0.001**	**0.010**	0.901	**0.042**
DHA	1.606± 0.157	2.119±0.147	1.512± 0.119	1.955± 0.148	1.443± 0.297	2.322±0.213	1.210± 0.199	1.908±0.170	**0.022**	**0.023**	**0.037**	**0.017**
Total PUFA n-3	0.698± 0.074	1.048± 0.100	0.642± 0.059	0.962± 0.080	0.703± 0.134	1.024± 0.176	0.568± 0.088	0.966± 0.141	0.061	**0.040**	0.178	0.112
LA	7.176± 0.453	6.002±0.300	6.982± 0.464	5.953± 0.321	8.040± 0.875	8.367±0.801	6.391± 0.467	6.336± 0.789	**0.037**	**0.048**	0.789	0.952
EDA	0.282±0.033	0.426± 0.051	0.214± 0.027	0.317± 0.032	0.430± 0.055	0.413± 0.033	0.238± 0.034	0.250± 0.037	**0.023**	**0.007**	**0.048**	0.812
DGLA	3.415± 0.525	4.547± 0.437	2.389± 0.245	4.258±0.358	2.536±0.823	5.026±0.666	2.367± 0.465	3.759±0.280	**0.023**	**<0.001**	**0.047**	**0.021**
ARA	12.495±0.592	10.827± 0.483	11.280± 0.593	9.658±0.573	9.502± 1.506	8.292± 0.859	12.548± 1.066	8.699± 1.044	**0.041**	**0.040**	0.501	**0.020**

DPA	0.157± 0.018	0.216±0.022	0.116± 0.022	0.164±0.019	0.115± 0.043	0.110± 0.017	0.147± 0.028	0.157± 0.027	**0.043**	**0.007**	0.589	0.929
AdA	1.869± 0.090	1.516±0.135	1.602± 0.106	1.288±0.115	1.755±0.224	1.083±0.172	1.747± 0.202	1.252±0.179	**0.039**	**0.049**	**0.038**	**0.031**
Total n-6 PUFA	4.407± 0.450	4.090±0.380	3.717± 0.353	3.591± 0.305	3.736± 0.709	3.849± 0.648	3.906± 0.635	3.409± 0.490	0.767	0.440	0.716	0.842

Note: Values are mean ± SEM. Bold values highlight statistical significance, while grey rows denote the main groups of fatty acids. *Abbreviations*: NT – normal tissue; EC – endometrial cancer; IA, IB, II, III – stages of EC; ND – not detected; *10:0 – capric acid; 12:0 – lauric acid; 14:0 – myristic acid; 16:0 – palmitic acid; 18:0 – stearic acid; 20:0 – arachidic acid; 22:0 – behenic acid; 24:0 – lignoceric acid; 26:0 – cerotic acid; 28:0 – montanic acid; Total ECFA – total even-chain fatty acids; 11:0 – undecanoic acid; 13:0 – tridecanoic acid; 15:0 – pentadecanoic acid; 17:0 – heptadecanoic acid; 19:0 – nonadecanoic acid; 21:0 – heneicosanoic acid; 23:0 – tricosanoic acid; 25:0 – pentacosanoic acid; Total OCFA – Total odd-chain fatty acids; Total SFA – Total saturated FA; iso 12–M–13:0 – iso-methyl branched 13:0 FA; iso 13–M–14:0 – iso-methyl branched 14:0 FA; iso 14–M–15:0 – iso-methyl branched 15:0 FA; iso 15–M–16:0 – iso-methyl branched 16:0 FA; iso 20–M–21:0 – iso-methyl branched 21:0 FA; iso 22–M–23:0 – iso-methyl branched 23:0 FA; Total iso BCFA – Total iso-branched-chain FA; anteiso 12–M–14:0 – anteiso-methyl branched 14:0 FA; anteiso 14–M–16:0 – anteiso-methyl branched 16:0 FA; Total anteiso BCFA – Total anteiso-branched-chain FA; 14:1 – myristoleic acid; 16:1 – palmitoleic acid; 17:1 – heptadecenoic acid; 18:1 – oleic acid; 19:1 – nonadecenoic acid; 20:1 – eicosenoic acid; 22:1 – erucic acid; 24:1 – nervonic acid; Total MUFA – Total monounsaturated FA; ALA – alpha-linolenic acid; ETA – eicosatrienoic acid; EPA – eicosapentaenoic acid; DPA – docosapentaenoic acid; DHA – docosahexaenoic acid; Total PUFA n-3 – Total omega-3 polyunsaturated FA; LA – linoleic acid; EDA – eicosadienoic acid; DGLA – dihomo-gamma-linolenic acid; ARA – arachidonic acid; AdA – adrenic acid; DPA – docosapentaenoic acid; Total PUFA n-6 – Total omega-6 polyunsaturated FA.*

Table S3. Primer sequences used for the determination of gene expression by RT-PCR

Target genes	Sequence R	Sequence F
Cyclophilin A	TCGAGTTGTCCACAGTCA	CGTCTCCTTTGAGCTGT
ELOVL1	CTGGGAGATGTGCAGTGAGA	CTGTGGCACAACCCTACCTT
ELOVL2	CCCAGCCATATTGAGAGCAGATA	ATGTTTGGACCGCGAGATTCT
ELOVL4	CACACGCTTATCTGCGATGG	GAGCCGGGTAGTGTCCTAAAC
FADS1	AAGAATTGCGTGTGCCAGGAC	TCAGTTTCAGAAGCAGGCAGGA
FADS2	GATTGTAGGGCAGGTATTTCAGC	AAGGGTGCCTCTGCCAACT
SCD1	GGTAGTTGTGGAAGCCCTC	AACAGTGTGTTCGTTGCCACTT
ACACA	GTTATCCCCAAACCCAGGCA	GCCTGACTTTTGATCCGACC
CD36	ACACAGCGTAGATAGACCTGC	TGGCCAAGCTATTGCGACAT
FASN	CTCGTTGAAGAACGCATCCA	CGCTCGGCATGGCTATCT
CPT1a	TCAGGGAGTAGCGCATGGT	ATCAATCGGACTCTGGAAACGG

Note: ELOVL1,2,4 – very long chain fatty acid elongase 1,2,4; FADS1,2 – fatty acid desaturase 1,2; SCD1 – stearoyl-CoA desaturase 1; ACACA - Acetyl-CoA Carboxylase; CD36 - fatty acid translocase; FASN - fatty acid synthase; CPT1a - carnitine palmitoyltransferase 1A

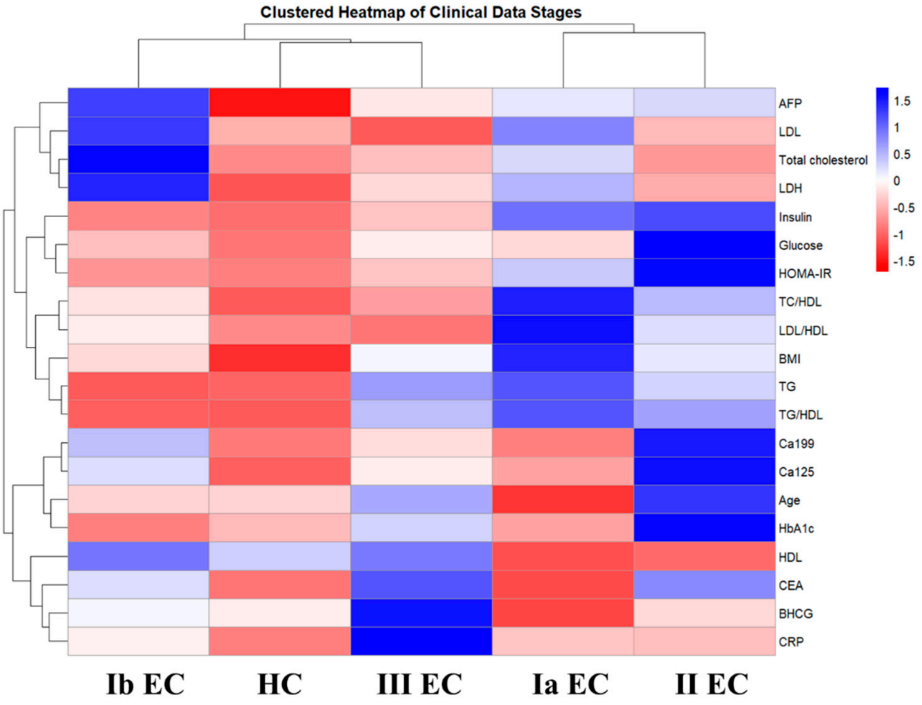

Figure S1. Heat map summarising clinical data of the study cohort (n=83)

This heat map illustrates clinical measurements across different stages of disease (EC Ia, Ib, II, III) and a control group (HC). Each row represents a clinical measurement, and each column represents a patient group. The color scale ranges from blue (higher values) to red (lower values). A detailed summary of clinical data and patient characteristics is given in Table 1.

Abbreviations: HC – healthy control, EC – endometrial cancer, BMI - body mass index; HbA1c - glycosylated haemoglobin A1c; CRP - C-reactive protein, AFP - alpha-fetoprotein; β-HCG - β-Human chorionic gonadotropin; CEA - carcinoembryonic antigen; Ca19-9 - carbohydrate antigen 19-9; Ca125 - cancer antigen 125; TC - total cholesterol; HDL- high-density lipoprotein cholesterol; LDL - low-density lipoprotein cholesterol; TG – triglycerides.

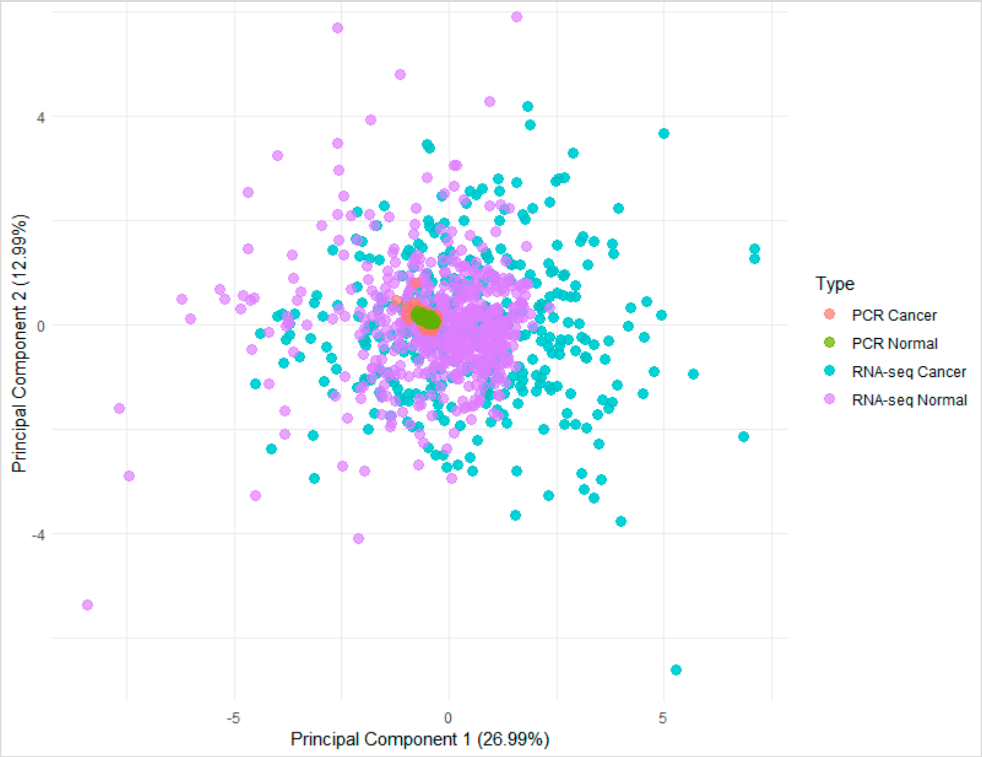

Figure S2. Principal Component Analysis (PCA) of RNA-seq and PCR data for cancer and normal tissues

The scatter plot represents the Principal Component Analysis (PCA) of RNA-seq and PCR data for both endometrial cancer and normal tissue samples. It displays the results of four datasets: PCR Cancer, PCR Normal, RNA-seq Cancer, and RNA-seq Normal. The x-axis (Principal Component 1) explains 26.99% of the total variance, while the y-axis (Principal Component 2) accounts for 12.99%. Different datasets are represented by different colours to allow visual differentiation and interpretation.

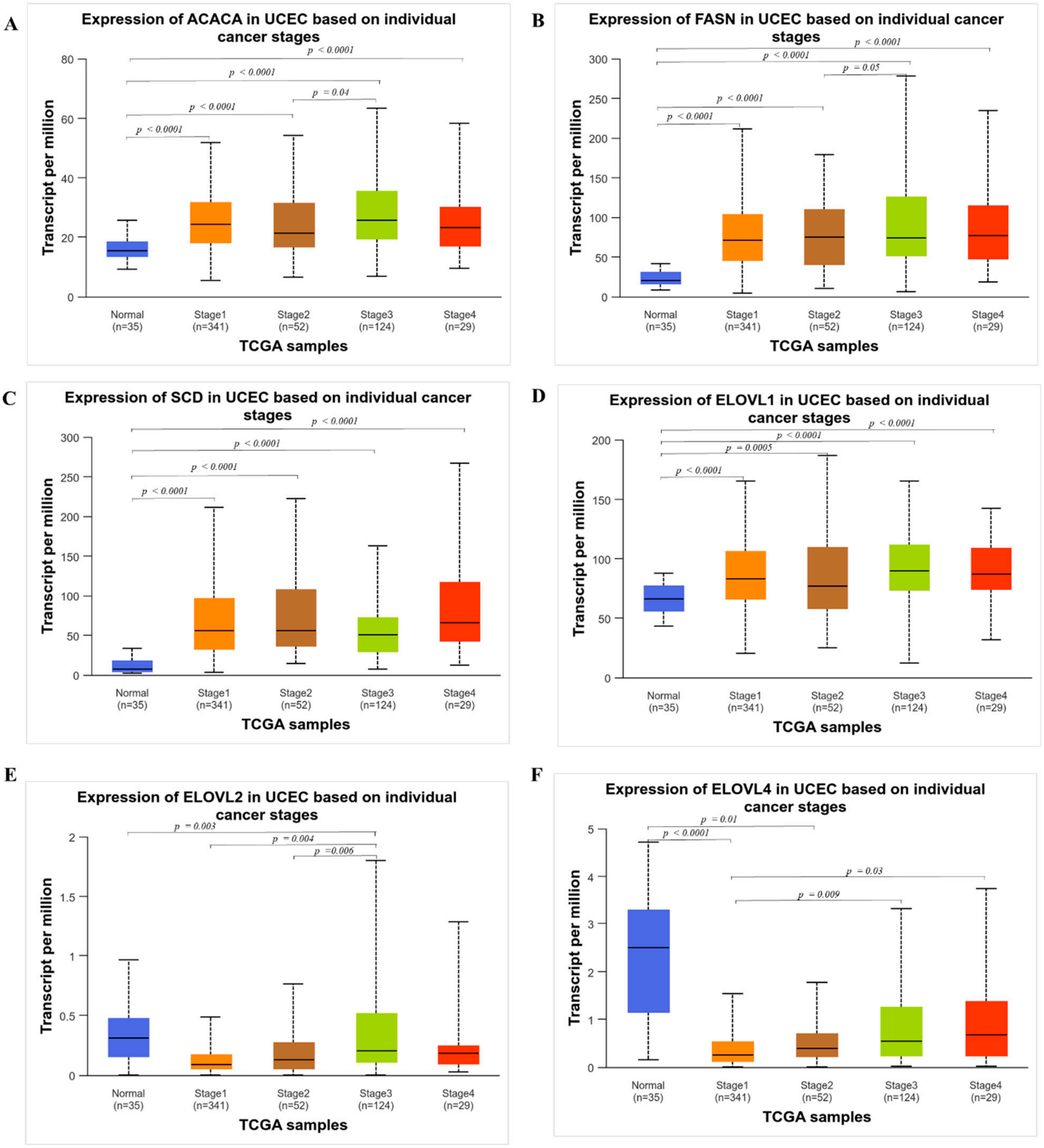
A
Expression of ACACA in UCEC based on individual cancer stages
p < 0.0001
p < 0.0001
p = 0.04
p < 0.0001
p < 0.0001
Transcript per million
Normal (n=35)
Stage1 (n=341)
Stage2 (n=52)
Stage3 (n=124)
Stage4 (n=29)
TCGA samples
B
Expression of FASN in UCEC based on individual cancer stages
p < 0.0001
p < 0.0001
p = 0.05
p < 0.0001
p < 0.0001
Transcript per million
Normal (n=35)
Stage1 (n=341)
Stage2 (n=52)
Stage3 (n=124)
Stage4 (n=29)
TCGA samples
C
Expression of SCD in UCEC based on individual cancer stages
p < 0.0001
p < 0.0001
p < 0.0001
p < 0.0001
Transcript per million
Normal (n=35)
Stage1 (n=341)
Stage2 (n=52)
Stage3 (n=124)
Stage4 (n=29)
TCGA samples
D
Expression of ELOVL1 in UCEC based on individual cancer stages
p < 0.0001
p < 0.0001
p = 0.0005
p < 0.0001
Transcript per million
Normal (n=35)
Stage1 (n=341)
Stage2 (n=52)
Stage3 (n=124)
Stage4 (n=29)
TCGA samples
E
Expression of ELOVL2 in UCEC based on individual cancer stages
p = 0.003
p = 0.004
p =0.006
Transcript per million
Normal (n=35)
Stage1 (n=341)
Stage2 (n=52)
Stage3 (n=124)
Stage4 (n=29)
TCGA samples
F
Expression of ELOVL4 in UCEC based on individual cancer stages
p = 0.01
p < 0.0001
p = 0.03
p = 0.009
Transcript per million
Normal (n=35)
Stage1 (n=341)
Stage2 (n=52)
Stage3 (n=124)
Stage4 (n=29)
TCGA samples

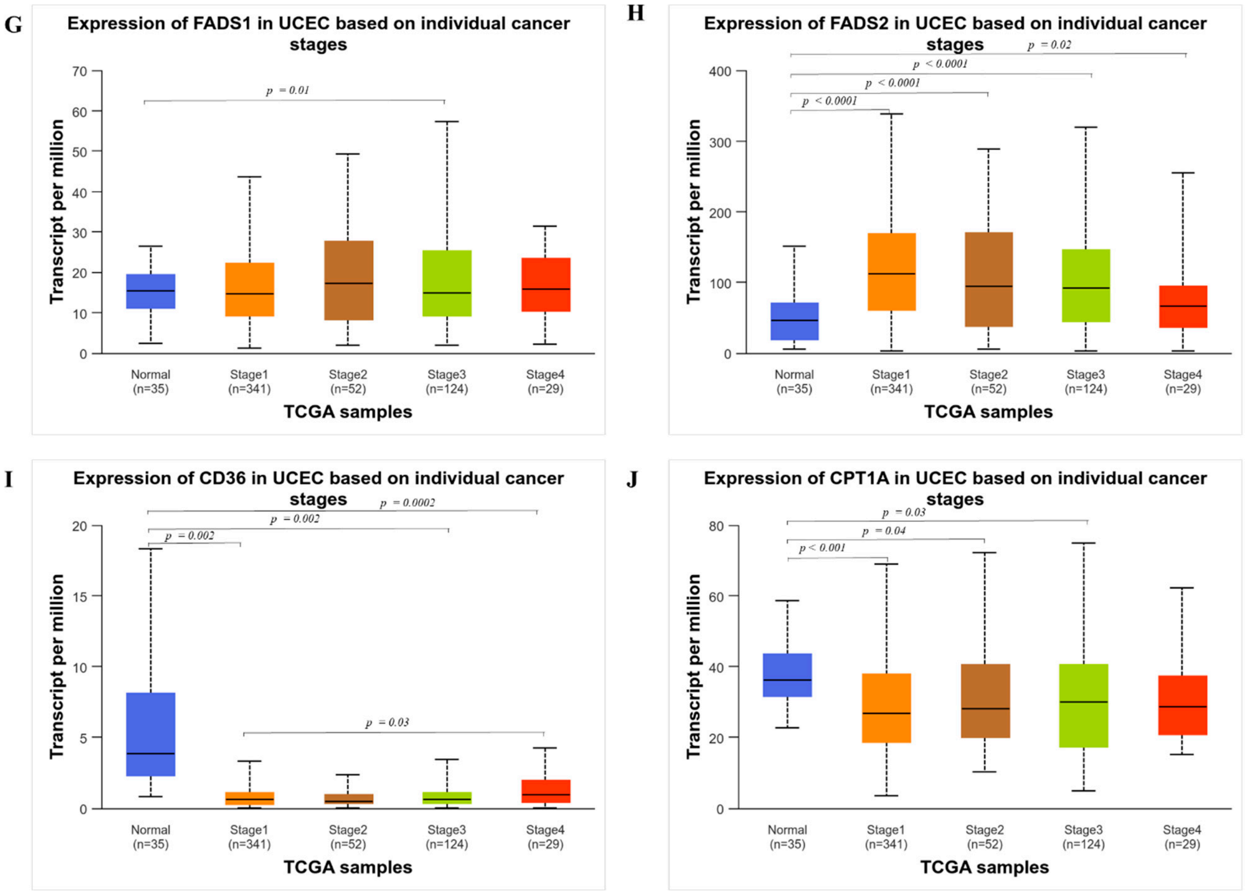

Figure S3. Expression patterns of *ACACA, FASN, SCD1, ELOVL1, ELOVL2, ELOVL4, FADS1, FADS2, CD36,* and *CPT1a* genes across Uterine corpus endometrial carcinoma (UCEC) stages based on TCGA data (1) (2)

The figure consists of box plots illustrating the expression levels of lipid metabolism-related genes across different stages of uterine corpus endometrial cancer (UCEC), based on RNA-seq data expressed as transcripts per million (TPM). The data, obtained from the TCGA UALCAN portal, are stratified into normal tissue samples (n=35) and cancer stages 1 to 4 (with sample sizes of n=341, n=52, n=124 and n=29, respectively). Box plots include statistical significance markers showing p-values for comparisons between groups.

Abbreviations: TCGA – the cancer genome atlas; ELOVL1,2,4 – very long chain fatty acid elongase 1,2,4; FADS1,2 – fatty acid desaturase 1,2; SCD1 – stearoyl-CoA desaturase 1; ACACA - Acetyl-CoA Carboxylase; CD36 - fatty acid translocase; FASN - fatty acid synthase; CPT1a - carnitine palmitoyltransferase 1A.

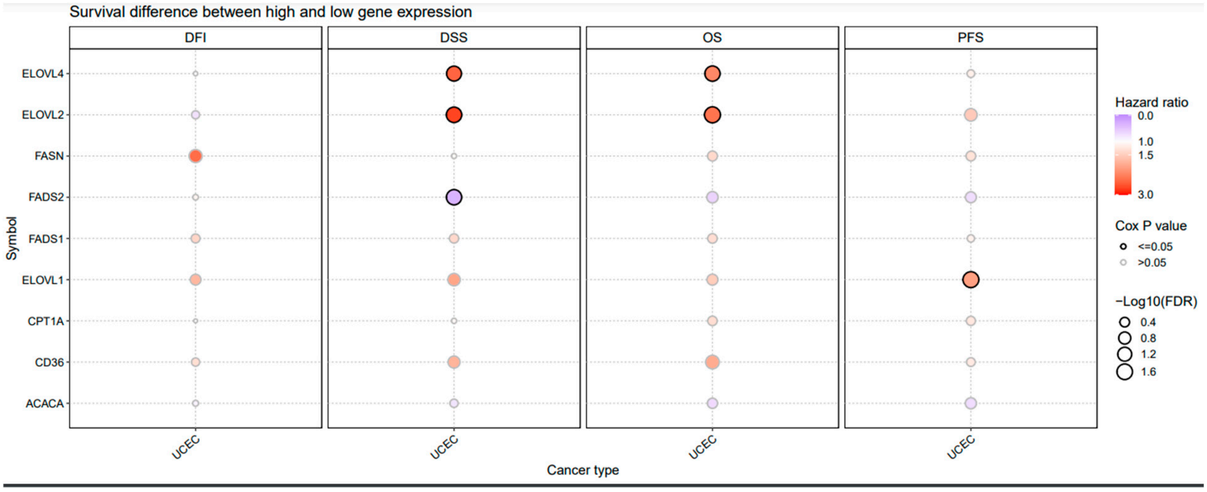

Figure S4. Summarizes the survival difference between high and low gene expression groups (3) (4)

This figure illustrates the association between the expression levels of several genes— *ACACA, FASN, ELOVL1, ELOVL2, ELOVL4, SCD1, FADS1, FADS2, CD36* and *CPT1a* —and survival outcomes in uterine corpus endometrial carcinoma (UCEC). Four different survival metrics are assessed: Disease-Free Interval (DFI), Disease-Specific Survival (DSS), Overall Survival (OS), and Progression-Free Survival (PFS).

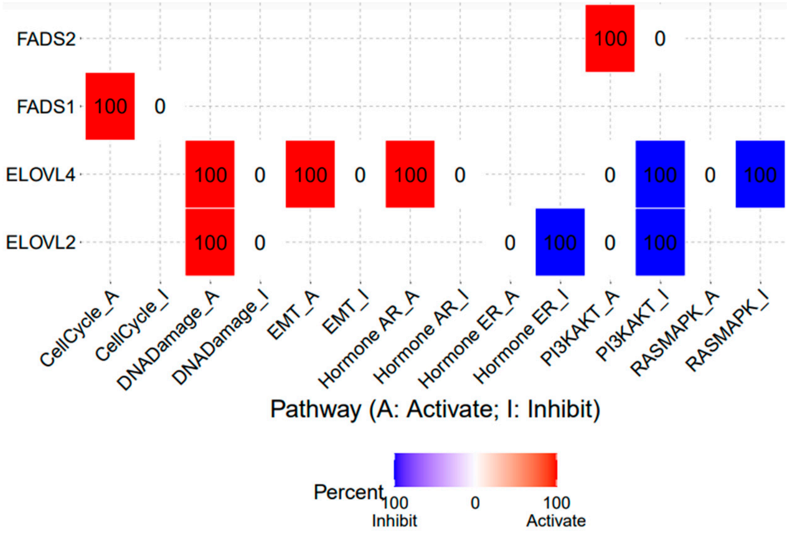

Figure S5. Influence of mRNA expression of lipid metabolism genes on pathway activity in uterine corpus endometrial carcinoma (3) (4)

This figure shows a summarizing the percentage of UCEC cases in which the mRNA expression of specific genes (FADS1, FADS2, ELOVL4, ELOVL2) has a potential effect on the activation or inhibition of various cellular pathways. The pathways are labeled on the x-axis, and the genes are listed on the y-axis. The color scale ranges from blue (indicating 100% inhibition) to red (indicating 100% activation).

Abbreviations: A – activation, I – inhibition, EMT - epithelial-to-mesenchymal transition, AR - androgen receptor, ER - estrogen receptor.

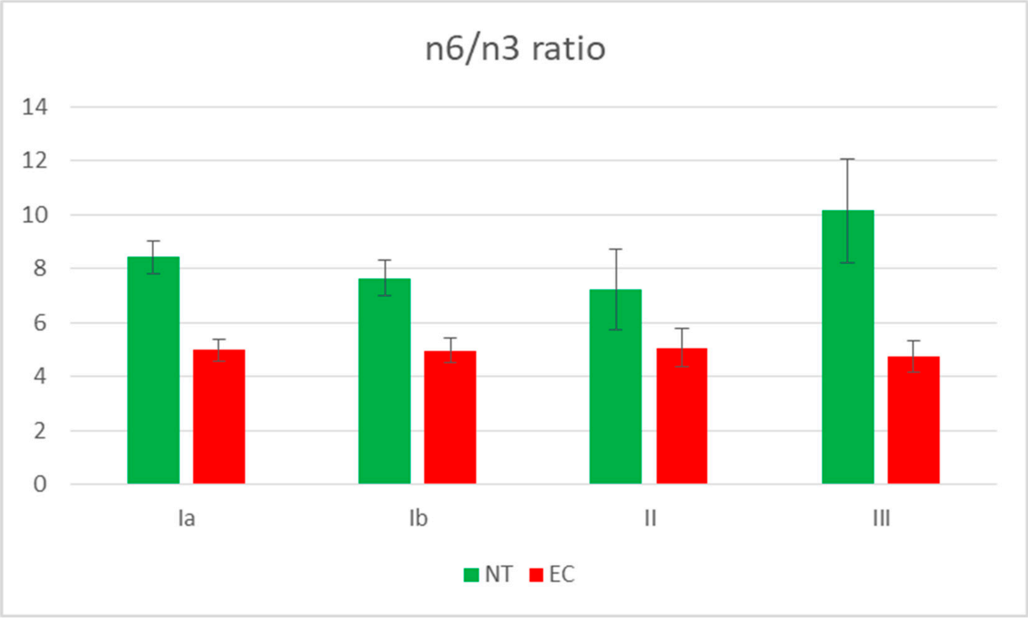

Figure S6. Comparison of n6/n3 ratio of PUFA in normal and cancerous endometrial tissues across different stages

Values are the mean ± SEM. This bar chart illustrates the comparison of the n6/n3 PUFA ratio in normal tissue (NT) and cancerous endometrial tissue (EC) in different groups labelled Ia, Ib, II and III, where n6 PUFA (ARA + EDA + DPA + DGLA + LA + AdA) and n3 PUFA (ALA + EPA + DHA + DPA).

Abbreviations: *ARA – arachidonic acid; EDA – eicosadienoic acid; DPA – docosapentaenoic acid; DGLA – dihomo-gamma-linolenic acid; LA – linoleic acid; AdA – adrenic acid; ALA – alpha-linolenic acid; EPA – eicosapentaenoic acid; DHA – docosahexaenoic acid; DPA – docosapentaenoic acid.*

1. Chandrashekar, D. S., S. K. Karthikeyan, P. K. Korla, H. Patel, A. R. Shovon, M. Athar, G. J. Netto, Z. S. Qin, S. Kumar, U. Manne, C. J. Crieghton, and S. Varambally. 2022. UALCAN: An update to the integrated cancer data analysis platform. *Neoplasia*. 25, 18–27.

2. Weinstein, J. N., E. A. Collisson, G. B. Mills, K. R. Mills Shaw, B. A. Ozenberger, K. Ellrott, I. Shmulevich, C. Sander, and J. M. Stuart. 2013. The Cancer Genome Atlas Pan-Cancer analysis project. *Nat. Publ. Gr.* 45, 1113 - 1120.

3. Liu, C. J., F. F. Hu, M. X. Xia, L. Han, Q. Zhang, and A. Y. Guo. 2018. GSCALite: a web server for gene set cancer analysis. *Bioinformatics*. 34, 3771–3772.

4. Liu, C. J., F. F. Hu, G. Y. Xie, Y. R. Miao, X. W. Li, Y. Zeng, and A. Y. Guo. 2023. GSCA: an integrated platform for gene set cancer analysis at genomic, pharmacogenomic and immunogenomic levels. *Brief. Bioinform.* 24.
